# Dietary phosphorus restriction induced phospholipid deficiency, endoplasmic reticulum stress, inflammatory response and gut microbiota disorders in *Lateolabrax maculatus*

**DOI:** 10.3389/fimmu.2025.1592806

**Published:** 2025-05-15

**Authors:** Zixiang Wu, Jiarong Guo, Kangle Lu, Kai Song, Ling Wang, Ruijuan Ma, Chunxiao Zhang, Xueshan Li

**Affiliations:** 1 State Key Laboratory of Mariculture Breeding, Fisheries College, Jimei University, Xiamen, China; 2 Xiamen Key Laboratory for Feed Quality Testing and Safety Evaluation, Fisheries College, Jimei University, Xiamen, China

**Keywords:** spotted seabass, low phosphorus diet, gut microbiota, lipid metabolism, inflammatory response, endoplasmic reticulum stress

## Abstract

This study evaluated the effects of low phosphorus on spotted seabass (*Lateolabrax maculatus*) from the perspective of phospholipid content and function, endoplasmic reticulum (ER) stress, inflammatory response and gut microbiota. Two diets were prepared to contain available phosphorus levels of 0.37% (low-phosphorus, LP) and 0.75% (normal-phosphorus, NP) and feed fish (3.53 ± 0.34 g) to satiety twice daily for 10 weeks. Compared with fish fed the NP diet, fish fed the LP diet showed lower body weight gain and higher abdominal fat percentage. Further studies showed that the LP diet decreased the content of phospholipid in the serum, liver, and abdominal fat tissue and induced ER stress and disruption of lipid metabolism in both of the liver and abdominal fat tissue and inflammatory responses in abdominal fat tissue. Furthermore, compared with fish fed the NP diet, the LP diet reduced microbial diversity in the gut. In contrast to fish fed the NP diet, fish fed the LP diet exhibited a decrease in the abundance of potential metabolically promoted probiotics (e.g., *Lactococcus lactis*) and an increase in the abundance of potential pathogenic bacteria (e.g., *Plesiomonas*) in the gut. The results of PICRUSt2 functional prediction also validated the metabolic disorders occurring in fish fed the LP diet as well as the reduced metabolic capacity. These results suggested that the LP diet decreased phospholipid content, induced ER stress and inflammatory responses then disturbed lipid metabolism and gut microbiota in spotted seabass. These negative effects contributed to poorer growth and higher percentage of abdominal fat in spotted seabass fed the LP diet than those of spotted seabass fed the NP diet.

## 1 Introduction

Being indispensable for animal physiology, phosphorus not only ensures proper bone development and maintenance but also drives fundamental biological processes including phospholipid production, genetic material synthesis, cellular communication, and systemic metabolism regulation (1–4). Teleosts primarily rely on dietary phosphorus due to limited physiological capacity to absorb dissolved phosphorus from water (5). The content of phosphorus in commercial feed is important for both the environment and economy (6). Phosphorus homeostasis in aquaculture systems presents critical environmental and physiological trade-offs. Excessive dietary phosphorus inputs contribute to aquatic eutrophication through effluent discharge (7), driving the aquaculture industry toward precision nutrition strategies. While the formulation of low-phosphorus (LP) feeds has gained prominence for ecological sustainability, insufficient phosphorus provision induces multisystemic dysfunction in aquatic species. Chronic phosphorus deficiency manifests as growth retardation, and metabolic dysregulation characterized by adipose tissue accumulation and skeletal mineralization defects (8, 9). As a critical regulatory factor in lipid metabolism, phosphorus modulates adipose deposition in fish (10, 11). Within intensive aquaculture systems, excessive abdominal fat accumulation reduces dress-out percentage and compromises feed conversion efficiency, thereby elevating production costs (12). Investigating the molecular interplay between phosphorus deficiency and lipid metabolism not only advances ecological conservation objectives but also establishes a theoretical foundation for precision nutritional management in aquaculture practices. Therefore, the role of phosphorus in various metabolic processes of organisms is worth investigating, which can contribute to the development of low-phosphorus feeds and few phosphorus emissions.

The endoplasmic reticulum (ER) serves as a central hub for multiple cellular processes, orchestrating calcium ion homeostasis, lipid metabolic regulation, and the synthesis, post-translational modification, and intracellular trafficking of proteins (13–15). In teleost species, phosphorus assumes critical importance in phospholipid biosynthesis, with the ER serving as the principal site for phospholipid anabolism. This organelle facilitates the enzymatic conversion of inorganic phosphorus into structural and functional phospholipid molecules through coordinated biochemical pathways (16). Phospholipids are essential components of the membranes of the ER and must be present in regular levels to maintain correct ER function (17). Abnormalities in the composition or fluidity of the ER membrane may compromise ER function and induce ER stress (18). ER stress can cause a range of negative effects such as insulin resistance, disruption of lipid metabolism as well as inflammatory responses (19–21). Furthermore, in mammals, the unfolded protein response (UPR) triggered by ER stress activates caspase-2-mediated NLRP3 inflammasome assembly (22), establishing a molecular bridge between ER stress and systemic inflammation. However, the association involving phosphorus and ER stress should be investigated further.

The vertebrate gastrointestinal tract constitutes a complex microbial ecosystem harboring dynamic symbiotic communities that exert essential influences on host nutritional assimilation and physiological homeostasis (23, 24). This intricate microbiota-host interface coordinates metabolic cross-talk through enzymatic diversification, micronutrient biosynthesis, and immunoregulatory signaling cascades. Many metabolic illnesses have been linked to alterations in the gut micro-ecosystem (25, 26). Dietary variables, such as macronutrients and micronutrients, can alter the composition and functionality of the gut microbiota (27). Phosphorus is necessary for both the host animal to maintain the normal metabolism and for the microbiota colonizing the animal’s GIT (28). The deficiency of phosphorus can reduce the ratio of probiotic/pathogenic bacteria (29). Emerging evidence underscores the critical role of probiotic microbiota in enhancing nutrient assimilation, preserving intestinal barrier integrity, and modulating immune responses (30, 31). Conversely, ecological perturbations within the intestinal microbiota significantly increase disease susceptibility, manifesting as metabolic dysregulation, chronic stress responses, and growth impairment (32, 33). Despite these advances, the mechanistic interplay between dietary phosphorus availability and microbial community dynamics remains poorly characterized, particularly in aquatic vertebrates.

The immune system employs both innate and adaptive mechanisms to maintain physiological homeostasis against pathogenic challenges (34, 35). Notably, nutritional imbalances can dysregulate NF-κB and JAK-STAT signaling pathways, compromising phagocytic activity of macrophages and neutrophil recruitment efficiency (36, 37). Emerging evidence from recent studies highlights the critical involvement of phospholipid metabolic homeostasis in modulating immune system functionality, underscoring its pivotal regulatory role in immunophysiological processes (38). However, the association involving phosphorus and ER stress should be investigated further, particularly regarding how phosphorus deficiency-induced phospholipid depletion modulates these inflammatory cascades in aquatic species.

The spotted seabass (*Lateolabrax maculatus*), a carnivorous teleost species widely farmed in China, exhibits distinct nutritional requirements for sustainable aquaculture. Our laboratory established 0.72% available phosphorus as the optimal dietary level for freshwater-reared specimens through rigorous dose-response trials (39). Strategic reduction of dietary phosphorus content presents a viable approach to mitigate aquaculture-derived phosphorus emissions, yet requires precise calibration to avoid compromising fish health and growth performance. However, the effects of low phosphorus diet on growth, metabolism and gut microbiota of spotted seabass remain to be studied. The present study is conducted to investigate the effects of low phosphorus on spotted seabass from the perspective of phospholipid content and function, ER stress, lipid metabolism and gut microbiota. This study also serves as a theoretical reference point for the application of low-phosphorus feeds and strategies to reduce phosphorus emissions.

## 2 Materials and methods

### 2.1 Animal policy and ethics

The study protocol received ethical approval (Permit number: 2011-58) from Jimei University’s Animal Ethics Review Board, with all procedures conducted in strict accordance with established animal welfare standards.

### 2.2 Diets and feeding experiment

Diets were formulated to contain either 0.37% available phosphorus (low-phosphorus, LP) or 0.75% available phosphorus (normal-phosphorus, NP). The specific composition and formulation details of these diets are documented in **Supplementary Table S1**. The feed preparation was informed by prior research conducted within our laboratory (40). Utilizing a recirculating aquaculture system (RAS) at Jimei University, the feeding trial was conducted in six 200-L fiberglass tanks. Healthy spotted seabass (*Lateolabrax maculatus*) with an initial mean body weight of 3.53 ± 0.34 g were procured from a commercial hatchery in Zhangzhou, Fujian Province, China. Following a 7-day acclimation period, 180 fish of uniform size were randomly allocated to the tanks (30 fish per tank). Fish were fed their respective experimental diets (LP or NP) to apparent satiation twice daily (08:00 and 17:00) for 10 weeks. The RAS maintained optimal water quality through continuous aeration and a 40% daily water exchange regimen. Key parameters were monitored and stabilized as follows: temperature 26–27°C, dissolved oxygen >6.5 mg/L, pH 6.9–7.2, and total ammonia nitrogen <0.2 mg/L. Tank assignments followed a completely randomized design to eliminate spatial bias.

### 2.3 Sample collection

After 24 hours of food deprivation, fish weight was measured to calculate growth metrics. Prior to tissue collection, fish were sedated with MS-222 anesthetic (60 mg/L solution, Sigma). From each tank, we obtained blood samples from 12 individuals through tail vein puncture using 27-gauge needles. These samples clotted overnight at 4 °C before centrifugation (1,283 × g, 10 minutes) to isolate serum for −80 °C storage. Three biological replicates were set up for each treatment in this study, and the samples from four fish were mixed together as one replicate to reduce variability between individual samples. Four jejunal samples were pooled per tank for microbiome analysis, with each specimen immediately flame-sterilized using an alcohol burner postcollection to mitigate cross-contamination risks. Liver, intestinal, and abdominal fat tissues were flash-frozen in liquid nitrogen and archived at −80°C for subsequent analyses.

### 2.4 Measures of biochemical parameters

Serum biochemical parameters, including alkaline phosphatase (ALP) activity, triacylglycerol (TG), phosphorus (P), and calcium (Ca) concentrations, were analyzed using commercial diagnostic kits (Jiancheng Bioengineering Institute, Nanjing, China). Quantification of phosphoglyceride (PG) and sphingomyelin (SM) levels in serum, hepatic, and adipose tissues was performed via enzyme-linked immunosorbent assay (ELISA) kits (Hengyuan Biotechnology, Shanghai, China). Liver enzymatic activities of choline phosphotransferase 1 (CHPT1) and ethanolamine phosphotransferase 1 (EPT1) were assessed using specific ELISA kits (Meimian Biological Technology, Yancheng, China).

### 2.5 Real-time quantitative PCR

Liver, jejunum, and abdominal fat tissue samples underwent triplicate RNA extraction using the established protocol from our established methodology (41). Subsequently, reverse transcription quantitative PCR (RT-qPCR) was performed on a QuantStudio 6 Pro system (Applied Biosystems) under optimized conditions: 95°C/10min initial denaturation, 40 cycles of 95°C/15s, and 60°C/1min. Primer design specifically targeted conserved regions within the spotted seabass transcriptome, with all oligonucleotides (**Supplementary Table S2**) exhibiting 90-110% amplification efficiency validated through standard curves (R²>0.99). Gene expression normalization employed the ΔΔCt algorithm, utilizing β-actin (CV<5% across biological replicates) as the endogenous control.

### 2.6 Liver histologic analysis and Oil Red O staining

Liver sections (5 μm) were cryosectioned and stained with H&E (5% acetic acid differentiation) and ORO (0.3% in isopropanol) following established protocols (42, 43). Bright-field imaging used a Leica DM5500B microscope (40×/NA 0.75) with Köhler illumination. For TEM, glutaraldehyde-fixed tissues were osmicated, dehydrated, and embedded in EPON 812 resin. Ultrathin sections (70 nm) stained with uranyl acetate/lead citrate were analyzed on a JEOL JEM-1400 TEM at 80 kV (44).

### 2.7 Illumina high-throughput sequencing

Jejunal microbial DNA was extracted using HiPure Soil DNA Kits (Magen Biotechnology) with bead-beating lysis. DNA quality (A260/A280 = 1.82 ± 0.05) was verified by NanoDrop 2000. The 16S rRNA V3-V4 regions were amplified with 338F/806R primers, purified using AxyPrep kits, and quantified via Qubit assays. Paired-end sequencing (2×250 bp) was performed on an Illumina MiSeq platform (GeneDenovo Biotechnology), yielding ~85,000 reads/sample.

Bioinformatics processing involved (1): Raw sequence demultiplexing and quality filtering using QIIME v1.9.1 with truncation parameters set at Q20 over 50-bp sliding windows (2); Chimera removal via reference-based detection using UCHIME algorithm against the SILVA 138 database (3); Operational Taxonomic Unit (OTU) clustering at 97% similarity threshold using UPARSE v7.1 (4); Taxonomic assignment based on Greengenes 13_8 reference database. Microbial community analyses included: α-diversity indices (Simpson and Pielou) calculation, β-diversity visualization through principal coordinates analysis (PCoA) based on Bray-Curtis dissimilarity using vegan v2.5-7, differential taxa identification via LEfSe (LDA score >2.0), and functional prediction through PICRUSt2 with KEGG pathway annotation. All visualizations were generated using ggplot2 v3.3.5 in R v4.1.2.

### 2.8 Statistical analysis

Statistical analyses were stratified by data type. For microbial community data, β-diversity dissimilarity matrices were subjected to permutational multivariate analysis (PERMANOVA) with 999 permutations using the Adonis function in the vegan package (v2.5.3). Differential taxa identification was performed through Welch’s two-sample t-tests on operational taxonomic units (OTUs) exhibiting >0.1% relative abundance. PICRUSt2-derived functional profiles and a-diversity indices (Shannon, Simpson) were compared between the two groups using Welch’s unequal variances t-test in R v4.1.2. Statistical analyses of non-microbiome data were performed using independent two-tailed t-tests in SPSS 25.0 (IBM, USA), with results presented as mean ± SEM. Significance thresholds were set at ns*P* ≥ 0.05, *P<0.05, **P<0.01, and ***P<0.001. In addition, the comparison of specific values in this paper follows the order of LP vs. NP, if there is no special explanation.

## 3 Result

### 3.1 Growth performance and abdominal fat percentage

**Figure 1:**
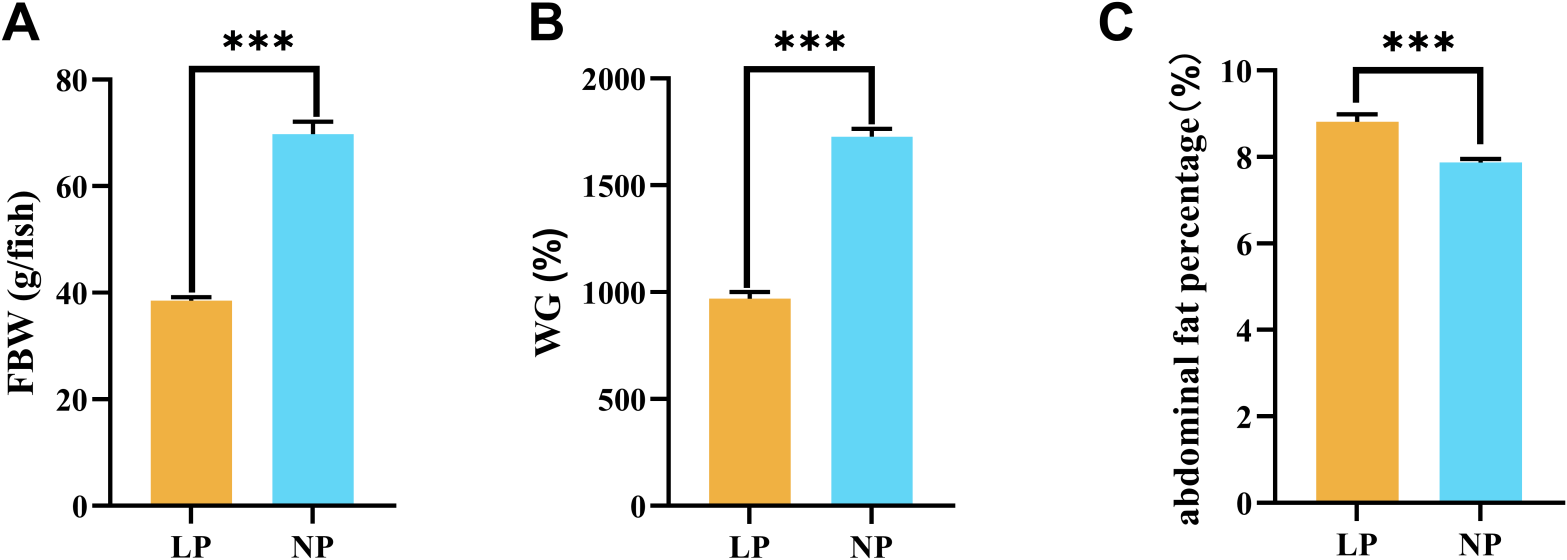
Comparative analysis of growth parameters in fish fed LP versus NP diets revealed distinct patterns in: **(A)** Final body weight; **(B)** Weight gain percentage*; **(C)** Abdominal fat percentage*. *Calculations: Weight gain (%) = [(Final - Initial body weight)/Initial body weight] × 100. Abdominal fat percentage (%) = (Abdominal fat mass/Final body weight) × 100. Data represent mean ± SEM values (n=9/group). Asterisks indicate statistically significant intergroup differences determined by two-tailed t-tests (***P < 0.001).

Fish fed the LP diet had a final body weight of 38.50 ± 0.66 g, representing a significant reduction compared to fish fed the NP diet (69.78 ± 2.29 g) (*P* < 0.001; **Figure 1A**). This growth retardation was further corroborated by weight gain (WG) metrics, where fish fed the LP diet (969.92 ± 17.91%) showed significantly lower values than fish fed the NP diet (1727.49 ± 21.42%) (*P* < 0.001; **Figure 1B**). However, fish fed the LP diet exhibited a significant increase in abdominal fat percentage (8.81 ± 0.10%) versus fish fed the NP diet (7.87 ± 0.05%) (*P* < 0.001; **Figure 1C**).

### 3.2 Phosphorus absorption and phospholipid content

**Figure 2:**
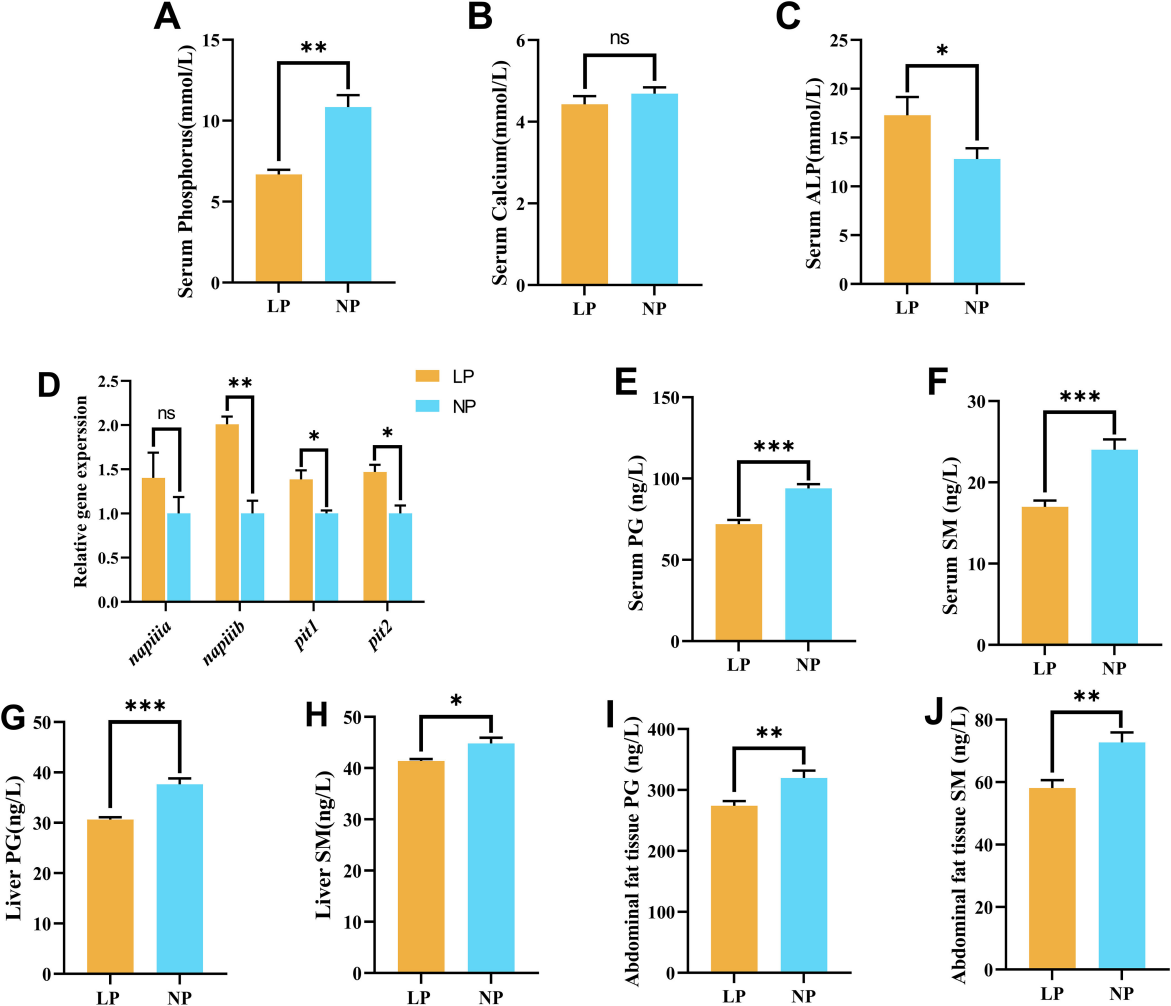
Comparative analysis of phosphorus metabolism biomarkers in fish fed LP versus NP diets revealed: **(A)** Serum phosphorus concentration; **(B)** Serum calcium level; **(C)** Alkaline phosphatase (ALP) activity; **(D)** Intestinal phosphorus transporter mRNA abundance*; **(E)** Serum phosphoglyceride (PG) content; **(F)** Serum sphingomyelin (SM) level; **(G)** Liver PG concentration; **(H)** Liver SM content; **(I)** Abdominal fat tissue PG level; **(J)** Abdominal fat tissue SM quantity. *Gene nomenclature detailed in **Supplementary Table S2**. Data represent mean ± SEM values (n=9/group) with asterisks indicating intergroup significance (*P<0.05, **P<0.01, ***P<0.001) determined by two-tailed independent t-tests. ns, non-significant.

Serum phosphorus concentrations in fish fed the LP diet (6.68 ± 0.29 mmol/L) exhibited a significant decrease compared to fish fed the NP diet (10.85 ± 0.73 mmol/L) (*P* < 0.01; **Figure 2A**), while serum calcium levels remained stable between the two groups (*P* = 0.366; **Figure 2B**). Concurrently, alkaline phosphatase activity in fish fed the LP diet (17.28 ± 1.09 mmol/L) showed a significant increase compared to fish fed the NP diet (12.79 ± 0.64 mmol/L) (*P* < 0.05; **Figure 2C**). Molecular analysis of intestinal transporters demonstrated phosphorus-specific regulation, with fish fed the LP diet displaying remarkable increases in *napi-iib*, *pit1*, and *pit2* genes expression compared to fish fed the NP diet (*P* < 0.05; **Figure 2D**). In contrast, *napi-iia* gene expression showed no significant difference (*P* = 0.304) between the two groups. Systemic phospholipid quantification identified consistent depletion patterns in fish fed the LP diet across all examined tissues. Phosphoglyceride (PG) and sphingomyelin (SM) contents in fish fed the LP diet were decreased in serum (PG: 71.94 ± 2.60 ng/L vs. 93.94 ± 2.60 ng/L, SM: 16.98 ± 0.78 ng/L vs. 24.00 ± 1.30 ng/L), liver (PG: 30.64 ± 0.47 ng/L vs. 37.63 ± 1.17 ng/L, SM: 41.38 ± 0.38 ng/L vs. 44.80 ± 1.14 ng/L), and abdominal fat tissues (PG: 274.12 ± 7.79 ng/L vs. 319.74 ± 12.12 ng/L, SM: 58.12 ± 2.48 ng/L vs. 72.69 ± 3.18 ng/L) compared to fish fed the NP diet, with all intergroup differences reaching statistical significance (*P* < 0.05; **Figures 2E–J**).

### 3.3 ER stress, lipid metabolism and inflammatory response

**Figure 3:**
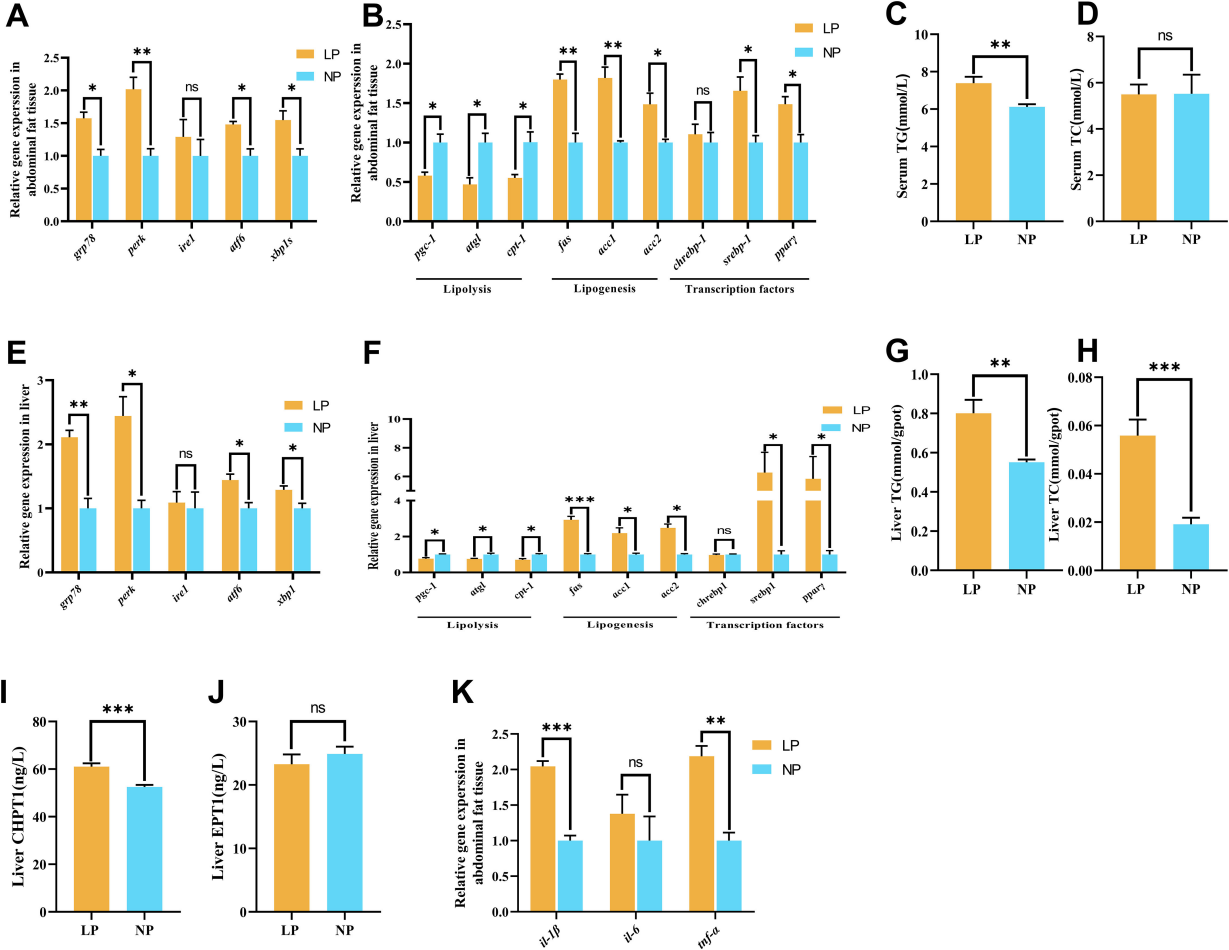
Comparative analysis of phosphorus metabolism biomarkers in fish fed LP versus NP diets revealed: **(A)** Abdominal fat tissue ER stress marker mRNA abundance; **(B)** Abdominal fat tissue lipid metabolism regulator expression; **(C)** Serum Triglyceride (TG) concentration; **(D)** Serum total cholesterol (TC) concentration; **(E)** Liver ER stress-related gene expression levels; **(F)** Liver lipid metabolism-related gene expression levels; **(G)** Liver TG concentration; **(H)** Liver TC content; **(I)** Liver choline phosphotransferase 1 (CHPT1) activity; **(J)** Liver ethanolamine phosphotransferase 1 (EPT1) activity; **(K)** Abdominal fat tissue pro-inflammatory cytokine levels. Gene nomenclature and inflammatory factor details are provided in **Supplementary Table S2**. Data represent mean ± SEM (n=9/group) with asterisks indicating significance (*P<0.05, **P<0.01, ***P<0.001) determined by two-tailed t-tests. ns, non-significant.

In abdominal fat and liver tissues, fish fed the LP diet exhibited a remarkable upregulation of ER stress related genes (*grp78*, *perk*, *atf6*, *xbp1s*) expression compared to fish fed the NP diet (*P* < 0.05; **Figures 3A, E**). Notably, *ire1* gene expression remained stable in both abdominal fat (*P* = 0.469) and liver (*P* = 0.774) tissues between the two groups.

Specifically, fish fed the LP diet showed a significant elevation with triacylglycerol (TG) content in serum (7.39 ± 0.20 mmol/L vs. 6.12 ± 0.08 mmol/L) and liver (0.80 ± 0.07 mmol/gpot vs. 0.55 ± 0.01 mmol/gpot) compared to fish fed the NP diet (*P* < 0.05; **Figures 3C, G**). Notably, liver total cholesterol (TC) content in fish fed the LP diet (0.056 ± 0.007 mmol/gpot) was significantly higher than fish fed the NP diet (0.019 ± 0.003 mmol/gpot) (*P* < 0.001; **Figure 3H**), while serum TC concentrations displayed no significant difference between the two groups (*P* = 0.970; **Figure 3D**). Intriguingly, coordinated lipid metabolic changes were observed across tissues. In both liver and abdominal fat tissue, fish fed the LP diet exhibited a remarkable downregulation of lipolysis genes (*pgc-1*, *atgl*, *cpt-1*) expression, whereas lipogenesis (*fas*, *acc1*, *acc2*) and regulatory factors (*srebp-1*, *pparγ*) expresssion were remarkably upregulated compared to fish fed the NP diet (*P* < 0.05; **Figures 3B, F**). Nevertheless, *chrebp-1* gene expression remained stable in both tissues (*P* = 0.598). Hepatic CHPT1 activity in fish fed the LP diet (61.06 ± 1.392 ng/L) was significantly increased compared to fish fed the NP diet (52.53 ± 0.869 ng/L) (*P* < 0.001; **Figure 3I**), contrasting sharply with unaltered EPT1 activity (*P* = 0.170; **Figure 3J**).

Fish fed the LP diet exhibited a remarkable upregulation of *il-1β* and *tnf-α* genes expression compared to fish fed the NP diet (*P* < 0.01). In contrast, *il-6* gene expression in abdominal fat tissue showed no remarkable difference between fish fed the LP and NP diets (*P* = 0.433; **Figure 3K**).

### 3.4 Histology of liver and abdominal fat tissue

**Figure 4:**
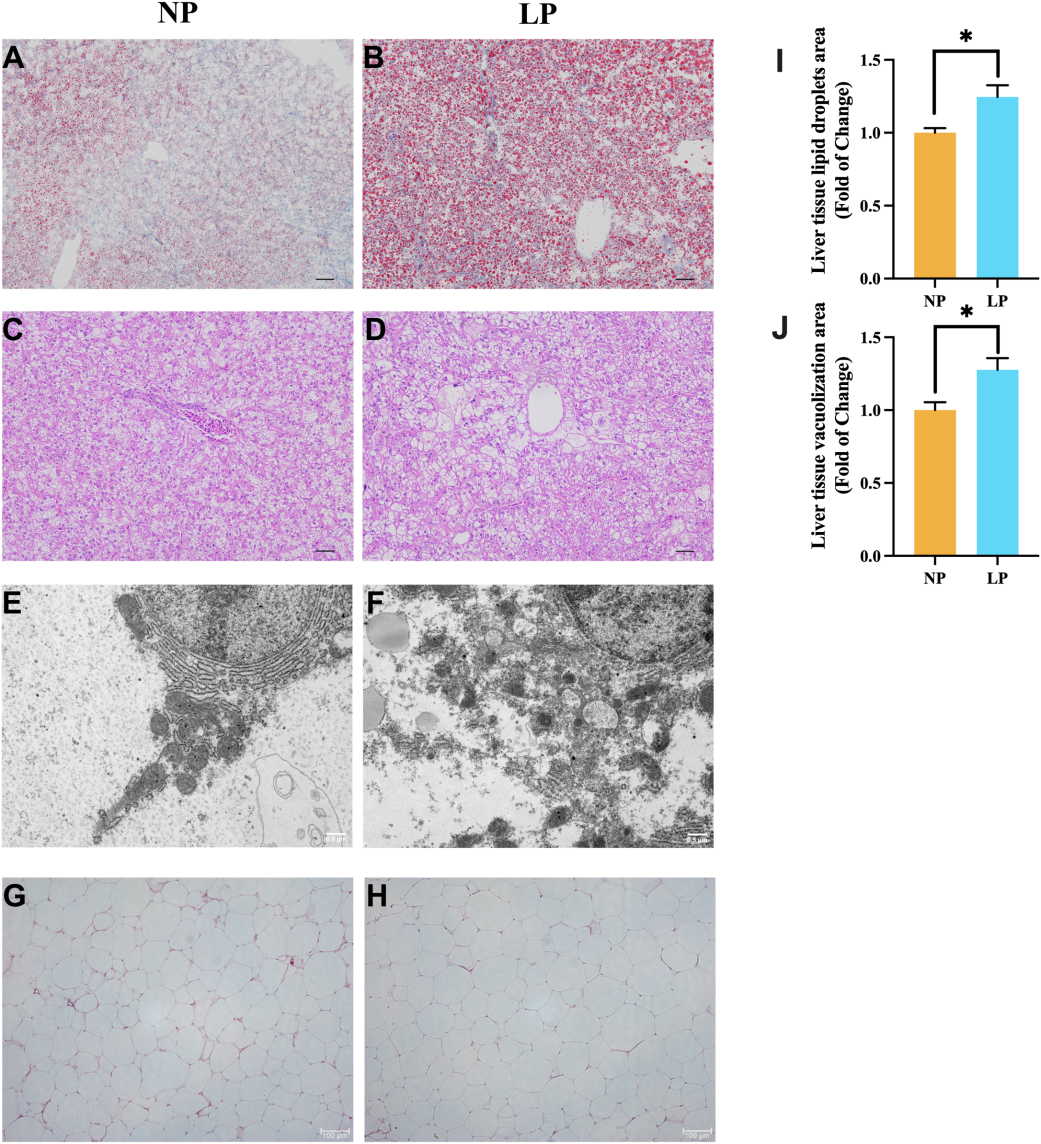
Comparative histopathological characterization of fish fed LP versus NP diet groups revealed: **(A, B)** Oil Red O-stained lipid deposition (Scale bar = 200 μm); **(C, D)** H&E-stained parenchymal architecture (Scale bar = 400 μm); **(E, F)** TEM visualization of endoplasmic reticulum (Original magnification ×7,000; Scale bar = 0.5 μm); **(G, H)** H&E-stained adipocyte morphology (Scale bar = 100 μm); **(I)** Vacuolization area of liver tissue (n = 3 fish, 3 visual fields/fish); **(J)** Lipid droplets area of liver tissue (n = 3 fish, 3 visual fields/fish). Data represent mean ± SEM (n=9/group) with asterisks indicating significance (*P < 0.05) determined by two-tailed t-tests.

The ORO sections revealed that the spotted seabass fed the LP diet had a greater fat accumulation in liver compared to fish fed the NP diet (*P* < 0.05; **Figures 4A** vs. **B, I**). In H&E sections, the vacuolization in liver of spotted seabass fed the LP diet was more serious than that of spotted seabass fed the NP diet (*P* < 0.05; **Figures 4C** vs. **D, J**). In addition, under the ultrastructure, the hepatocyte endoplasmic reticulum structure of spotted seabass fed the LP diet was severely damaged, the endoplasmic reticulum was loosely stacked, and the mitochondria-associated membranes (MAMs) was disorganized (**Figures 4E** vs. **F**). Meanwhile, the abdominal fat tissue of spotted seabass fed the LP diet showed adipocyte hypertrophy under H&E staining (**Figures 4G** vs. **H**).

### 3.5 Gut bacterial communities

**Figure 5:**
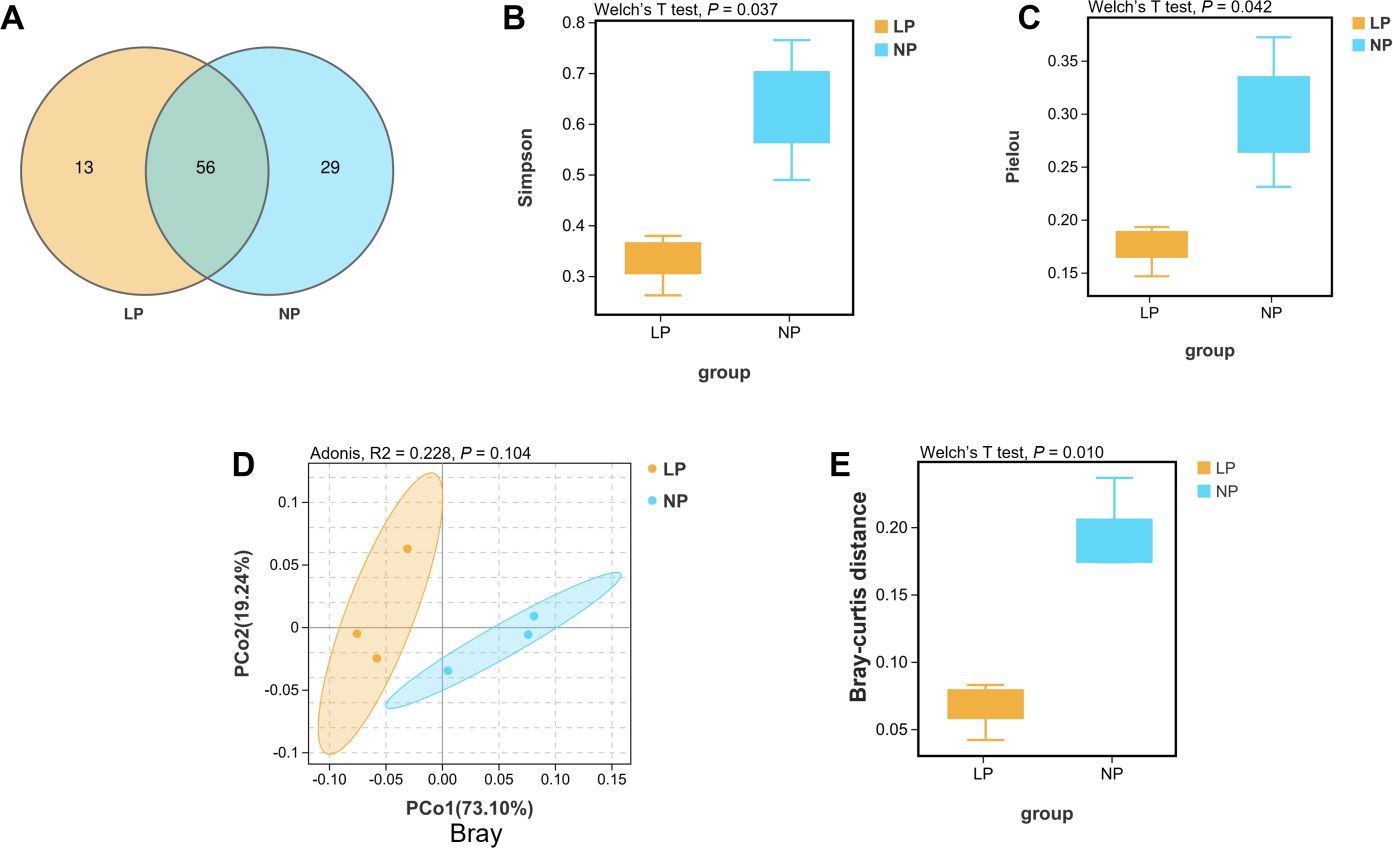
Comparative 16S rRNA phylogenetic profiling of fish fed LP versus NP diet groups revealed: **(A)** Venn diagrams comparing the OTUs of gut bacterial communities. **(B, C)** Alpha diversity evaluation using the Simpson and Pielou indices. **(D, E)** Bray-Curtis distance and corresponding beta diversity index results for the gut microbiota, analyzed with Adonis and Welch’s t-test.

Venn diagram quantification identified 56 shared operational taxonomic units (OTUs) between the two groups, with fish fed the LP diet harboring 13 unique OTUs compared to 29 in fish fed the NP diet (**Figure 5A**). Alpha diversity metrics demonstrated significantly reduced community heterogeneity in fish fed the LP diet, exhibiting markedly lower Simpson index and markedly decreased Pielou evenness relative to fish fed the NP diet (*P* < 0.05; **Figures 5B, C**). Multivariate analysis confirmed distinct clustering patterns, with principal coordinates analysis (PCoA) based on Bray-Curtis distances revealing remarkable separation between gut microbiota profiles of fish fed the LP and NP diets (*P* < 0.05; **Figures 5D, E**).

**Figure 6:**
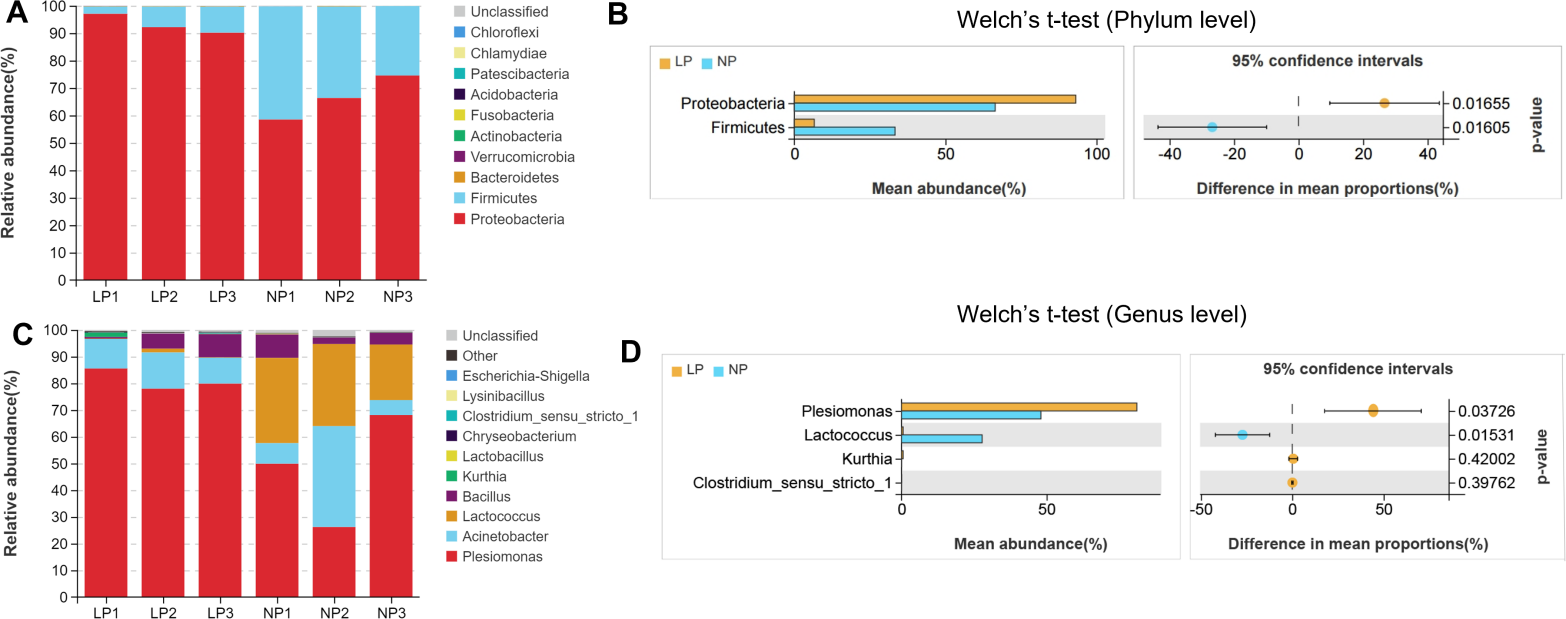
Comparative taxonomic stratification of gut microbiota infish fed LP versus NP diet groups revealed:Phylum-Level Profiling; **(A)** Stacked bar chart of bacterial composition; **(B)** Phylum abundance distribution; **(C)** Taxonomic composition bar plot; **(D)** Genus abundance comparison. Differences were assessed using Welch’s t-test.

At the phylum level, *Proteobacteria* and *Firmicutes* dominated intestinal communities across both dietary regimens. Fish fed the LP diet exhibited markedly higher relative abundance of Proteobacteria (93.12% vs. 66.47%) and markedly lower *Firmicutes* representation (6.56% vs. 33.31%) compared to fish fed the NP diet (*P* < 0.05; **Figures 6A, B**).

At the genus level, fish fed the LP diet were dominated by the bacterial taxa *Plesiomonas* (81.13%), *Acinetobacter* (11.45%), and *Bacillus* (4.96%) (**Figure 6C**). In fish fed the NP diet, *Plesiomonas* (48.08%), *Lactococcus* (27.82%), *Acinetobacter* (17.02%) and *Bacillus* (5.16%) were dominant bacterial taxa. Potential pathogenic bacteria, e.g. *Plesiomonas*, was significantly more abundant (*P* < 0.05), while the abundance of potential probiotics, e.g. *Lactococcus*, were significantly lower in fish fed the LP diet compared to those fed the NP diet (*P* < 0.05; **Figure 6D**).

**Figure 7:**
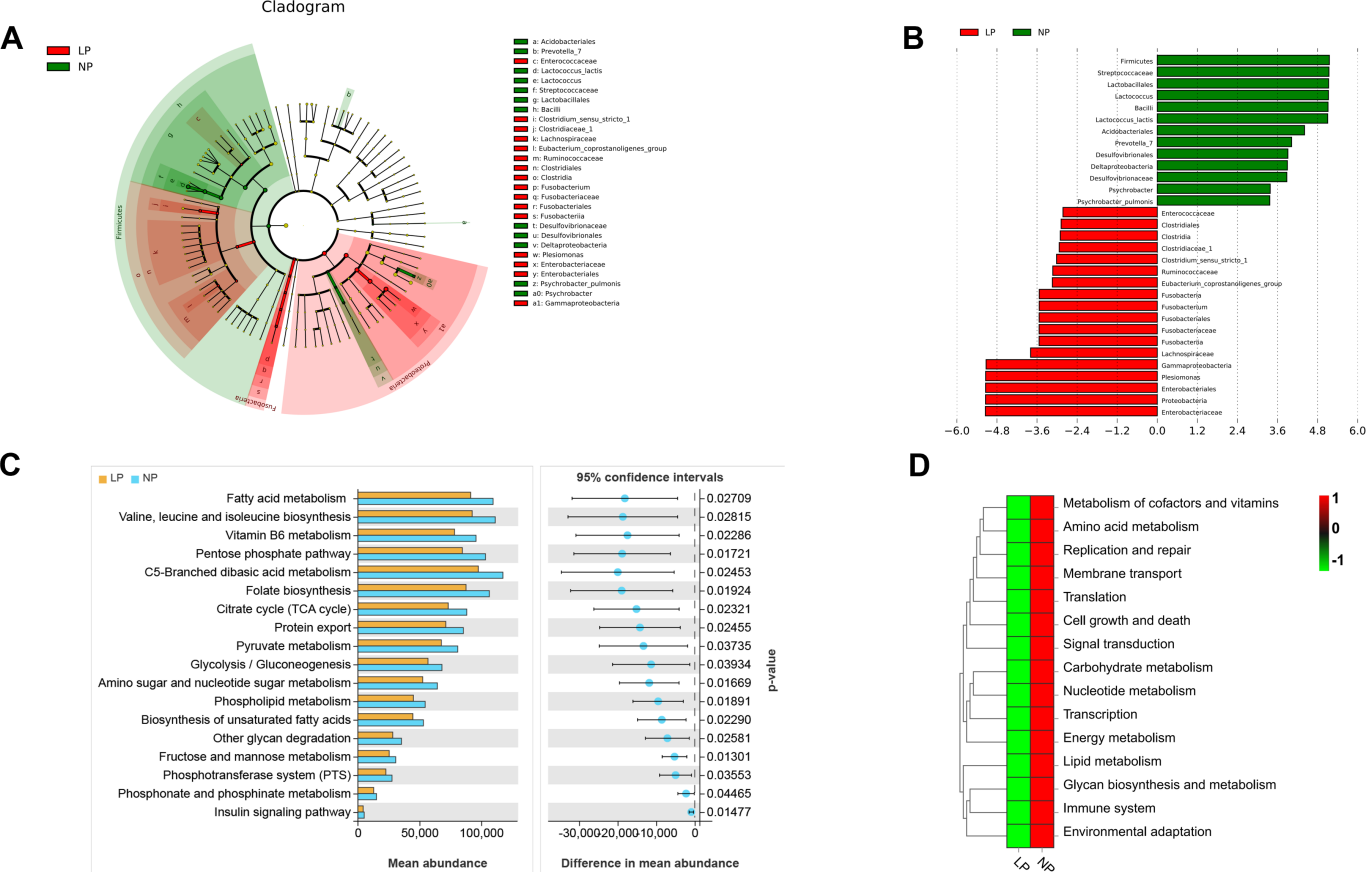
Functional metagenomic characterization of gut microbiota in LP *vs* NP diet groups: **(A)** LEfSe cladogram highlighting phylogenetic disparities (LDA score >3.5); **(B)** PICRUSt2-predicted MetaCyc pathway enrichment patterns; **(C)** KEGG level-2 pathway differential abundance (Welch’s t-test, FDR<0.05) **(D)** Heatmap visualization of level-3 enzymatic activity variations. Differences were calculated using Welch’s t-test.

LEfSe analysis revealed that fish fed the LP diet had significantly higher levels of *Plesiomonas*, *Ruminococcaceae*, *Gammaproteobacteria*, *Lachnospiraceae*, *Fusobacteriales*, *Enterobacteriales*, and *Clostridium_sensu_stricto_1*. In contrast, fish fed the NP diet had significantly enriched levels of *Lactococcus*, *Desulfovibrionales*, *Lactococcus_lactis*, *Bacillus*, *Lactobacillales*, *Streptococcaceae*, *Acidobacteriales*, and *Prevotella_7* compared to fish fed the LP diet (*P* < 0.05; LDA > 3.6; **Figures 7A, B**).

At KEGG hierarchy levels 2 and 3, 13 pathways were significantly enriched in fish fed the NP diet compared to fish fed the LP diet (*P* < 0.05). These encompassed lipid metabolism modules (fatty acid/phospholipid/glycerolipid metabolism, unsaturated fatty acid biosynthesis), energy transduction systems (insulin signaling), and membrane transport mechanisms (phosphotransferase system). Additional enriched pathways spanned carbohydrate metabolism (citrate cycle, pyruvate metabolism), cofactor/vitamin processing (vitamin B6), amino acid biosynthesis (valine-leucine-isoleucine), cellular homeostasis (growth/death, replication/repair), signal transduction cascades, glycan biosynthesis, environmental adaptation, translational machinery, and immune regulation (**Figures 7C, D**).

## 4 Discussion

Based on our laboratory’s prior research establishing 0.72% available phosphorus (NP) as the appropriate dietary phosphorus level for *Lateolabrax maculatus*, whereas 0.37% available phosphorus (LP) demonstrated a significant deficiency relative to the optimal value for investigating phosphorus deprivation effects, two experimental diets were formulated accordingly (39). This experimental design specifically replicated these established available phosphorus concentrations (0.75% NP vs. 0.37% LP) to systematically examine phosphorus deficiency manifestations. Consistent with established nutritional physiology paradigms (45, 46), fish fed the LP diet exhibited significantly poorer growth performance and elevated abdominal fat deposition compared to fish fed the NP diet. Serum phosphorus levels were markedly reduced in fish fed the LP diet, while alkaline phosphatase (ALP) activity showed a significant elevation, a biochemical pattern aligning with observations in phosphorus-deficient teleosts (47–49). This profile reflects enhanced osteoblastic activity under phosphorus restriction, as previously documented in mammalian models (50). Molecular analysis revealed a significant upregulation of intestinal sodium-phosphate cotransporter genes (*napi-iib*, *pit1*, *pit2*) expression in fish fed the LP diet compared to fish fed the NP diet, consistent with the canonical phosphorus absorption pathway mediated by Na-Pi transporters (NaPi-IIa/b/c, PIT1/2) (51). These transcriptional adjustments mirror compensatory mechanisms observed in terrestrial vertebrates under phosphorus scarcity (52, 53), suggesting evolutionary conservation of adaptive responses to dietary phosphorus insufficiency.

Phosphorus distribution in teleosts follows conserved physiological patterns, with the majority of bodily phosphorus sequestered in mineralized tissues as hydroxyapatite [Ca_10_(PO_4_)_6_;(OH)_2_] (54). The liver and adipose tissue are central hubs for systemic lipid metabolism in teleosts. The liver orchestrates *de novo* lipogenesis and phospholipid synthesis, while adipose tissue serves as the primary site for lipid storage and mobilization (55–57). Phospholipid-bound phosphorus plays critical roles in biological membrane architecture, functional maintenance, and metabolic regulation (58, 59). These amphipathic molecules, classified into phosphoglycerides (PG) and sphingomyelins (SM) based on backbone structures (60), are principally synthesized in the endoplasmic reticulum (ER) where they maintain ER structural integrity (61). Their compositional variations directly modulate membrane fluidity, protein-lipid interactions, and vesicular trafficking (62), with emerging evidence linking phospholipid metabolism to ER stress responses (63, 64). Experimental data revealed systemic phospholipid depletion in fish fed the LP diet compared to fish fed the NP diet, with PG and SM levels significantly reduced in serum, liver, and abdominal fat tissue. Concurrently, ER stress related genes (*grp78*, *perk*, *atf6*, *xbp1s*) expresssion showed a significant upregulation in fish fed the LP diet versus fish fed the NP diet, suggesting phospholipid insufficiency-induced ER membrane destabilization.

Interestingly, CHPT1 activity, catalyzing the terminal Kennedy pathway step crucial for phospholipid homeostasis (65), was significantly elevated in fish fed the LP diet. This elevation coincided with ER stress activation, mirroring mammalian models where *xbp1*-mediated pathways regulate *chpt1* expression under ER stress (66). These observations suggest a potential compensatory mechanism wherein phospholipid biosynthesis is upregulated to mitigate LP diet-induced ER stress.

Evidence in the literature showed that ER stress could induce disturbed lipid metabolism, which resulted in abnormal fat deposition in the organism (67). Furthermore, ER stress could promote the entry of *srebp1c* into the nucleus and activate the expression of lipid synthesis-related genes (*fas* and *acc*) expression (68). *Xbp1* promoted the expression of lipid synthesis transcription factors *pparγ* (69), and *atf6* activation could also promote TG synthesis by increasing *fas* and *acc2* activity (70). In the current study, fish fed the LP diet exhibited higher serum TG level, increased expression of lipogenesis-related genes (*fas*, *acc1*, *acc2*) and key transcription factors of lipid metabolism (*srebp-1* and *pparγ*), along with lower expression of lipolysis-related genes (*pgc-1*, *atgl*, and *cpt-1*) compared to fish fed the NP diet. As a result, the alterations in lipid metabolism observed in fish fed the LP diet are likely a consequence of ER stress.

Moreover, our experiment found that the expression of inflammatory factors in the abdominal fat tissues of spotted seabass fed the LP diet was upregulated compared to those fed the NP diet. ER stress was closely related to the inflammatory response (71). PERK triggers the translocation of NF-κB into the nucleus, leading to the transcription of various inflammatory factors, such as *il-1β* and *tnf-α* (72). In this experiment, the observed decreased growth performance in spotted seabass fed the LP diet was probably due to ER stress resulting from impaired phospholipid synthesis, which subsequently triggered elevated inflammatory responses.

Dietary phosphorus availability exerts profound influence on gut microbial ecosystems, as nutritional substrates directly shape microbial community structure (73). Phosphorus’s essential role in microbial proliferation was firstly demonstrated in rumen microbiota studies (74, 75), with subsequent research confirming its regulatory effects on fish intestinal microbiomes (76). Under the current experimental conditions, fish fed the LP diet exhibited reduced gut microbial diversity and ecological destabilization compared to fish fed the NP diet.

Significant decreases in operational taxonomic unit richness and alpha diversity indices were observed in fish fed the LP diet compared to those fed the NP diet through microbial community analysis. Such microbial simplification has been epidemiologically linked to metabolic dysregulation and increased pathogenic colonization risks across vertebrate taxa (77, 78). Multivariate analysis through principal coordinates (PCoA) confirmed distinct clustering patterns between the two groups, indicating phosphorusdependent microbiome restructuring.

At the phylum level, *Proteobacteria* and *Firmicutes* dominated intestinal communities in both groups, aligning with teleost gut microbiota baselines (79, 80). However, genus-level shifts emerged under phosphorus restriction: fish fed the LP diet showed significant reduction in *Lactococcus* abundance and marked elevation of *Plesiomonas* compared to fish fed the NP diet. LEfSe biomarker analysis corroborated these compositional changes.

The microbial profile alterations carry functional implications. *Plesiomonas*, identified as a potential opportunistic pathogen in aquatic species (81), may compromise intestinal barrier integrity and large-scale death of aquatic animals. As a Gram-negative bacterium, its surface contains lipopolysaccharide (LPS), which has been extensively documented to induce intestinal immune dysregulation (82, 83). Conversely, *Lactococcus lactis* demonstrates probiotic properties through growth promotion and pathogen inhibition (84–86), with proven capacity to modulate intestinal immunity. This dual shift, pathogenic proliferation combined with probiotic depletion, likely disrupt the intestinal mucosal immunity of fish fed the LP diet and contribute to the growth retardation observed in fish fed the LP diet.

The gut microbiota functions as a symbiotic metabolic interface, critically modulating host nutrient processing and homeostasis (87). Functional metagenomic prediction revealed significant depletion of lipid and phospholipid metabolic pathways in fish fed the LP diet compared to fish fed the NP diet, aligning with observed systemic lipid dysregulation. Concurrent reductions occurred in carbohydrate metabolism, amino acid cycling, energy transduction, and vitamin processing pathways – all essential for organismal growth and development.

This microbial metabolic impairment corresponds with physiological observations, as optimal microbiota composition enhances host nutrient assimilation and metabolic efficiency (88, 89). Notably, the reduced abundance of *Lactococcus lactis* in fish fed the LP diet versus fish fed the NP diet can compromise nutrient bioavailability, given this species’ documented capacity to upregulate intestinal growth factors and nutrient absorption mechanisms (84, 85). These collective microbial shifts likely contribute to the metabolic inefficiency and growth retardation observed under phosphorus restriction. Although this study identified phospholipid synthesis limitations caused by low phosphorus levels, it still lacks in-depth exploration of specific aspects such as the exact affected types of phospholipids and the precise mechanisms triggering endoplasmic reticulum (ER) stress. Future experiments will further investigate the specific phospholipid changes induced by low phosphorus and the regulated mechanisms in ER.

## 5 Conclusion

**Figure 8:**
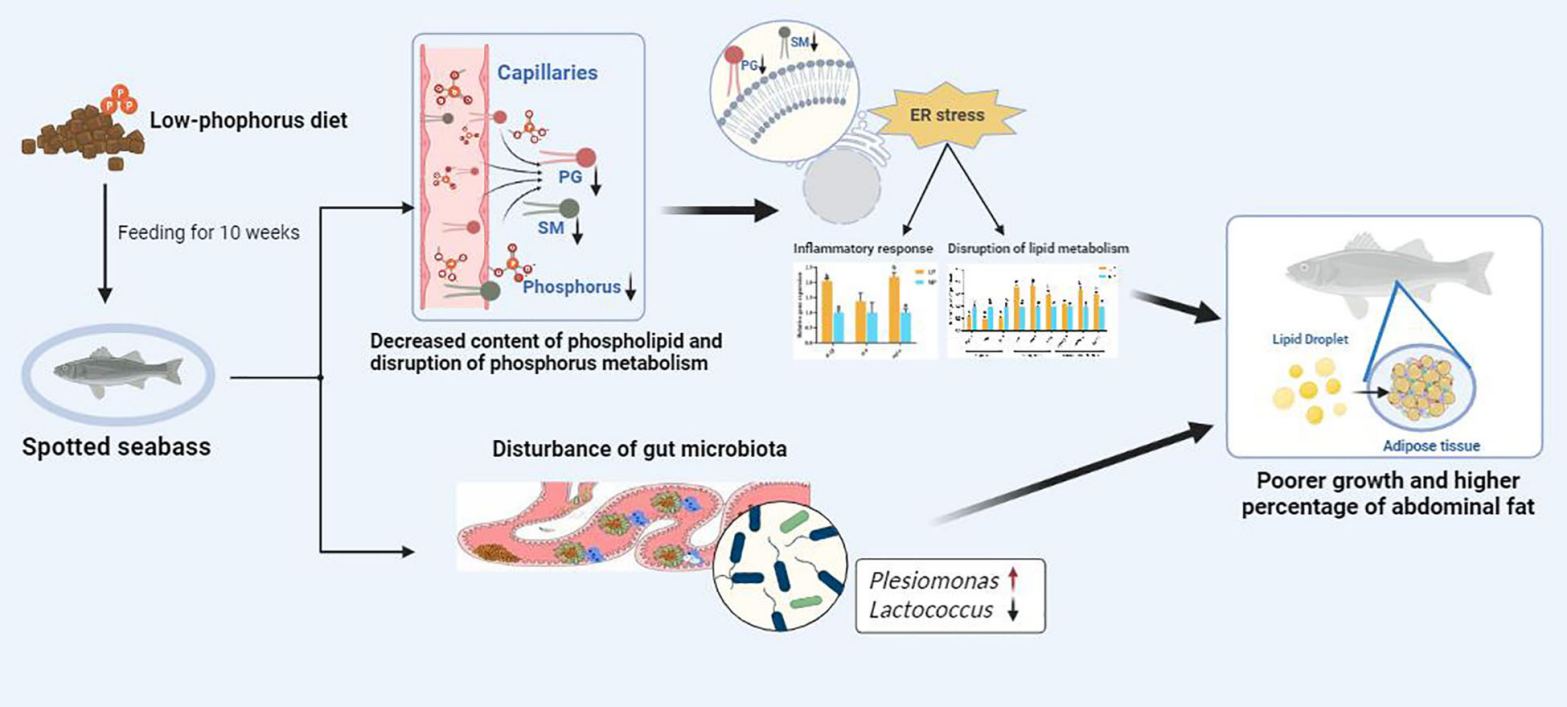
LP led to decreased content of phospholipid, ER stress, inflammatory responses and disruption of lipid metabolism as well as gut microbiota. These negative effects contributed to poorer growth and higher percentage of abdominal fat in spotted seabass fed the LP diet.

In this study (**Figure 8**), LP led to the decreased content of phospholipid in spotted seabass, which in turn induced ER stress, disturbed lipid metabolism and inflammatory response. Additionally, the LP diet resulted in reduced microbial diversity and modifications in the gut microbiota composition, thereby compromising intestinal immune competence. These negative changes likely contributed to the poorer growth and higher abdominal fat percentage observed in spotted seabass fed the LP diet.

## Data Availability

The original contributions presented in the study are included in the article/**Supplementary Material**. Further inquiries can be directed to the corresponding author.
