# Correction: Dietary phosphorus restriction induced phospholipid deficiency, endoplasmic reticulum stress, inflammatory response and gut microbiota disorders in *Lateolabrax maculatus*

**DOI:** 10.3389/fimmu.2025.1691775

**Published:** 2025-12-04

**Authors:** Zixiang Wu, Jiarong Guo, Kangle Lu, Kai Song, Ling Wang, Ruijuan Ma, Chunxiao Zhang, Xueshan Li

**Affiliations:** 1 State Key Laboratory of Mariculture Breeding, Fisheries College, Jimei University, Xiamen, China; 2 Xiamen Key Laboratory for Feed Quality Testing and Safety Evaluation, Fisheries College, Jimei University, Xiamen, China

**Keywords:** spotted seabass, low phosphorus diet, gut microbiota, lipid metabolism, inflammatory response, endoplasmic reticulum stress

There were errors in the legends for **Figure 1** and **Figure 4** as published; specifically, the significance descriptions were incorrect. The corrected legends appear below.

**Figure 1:**
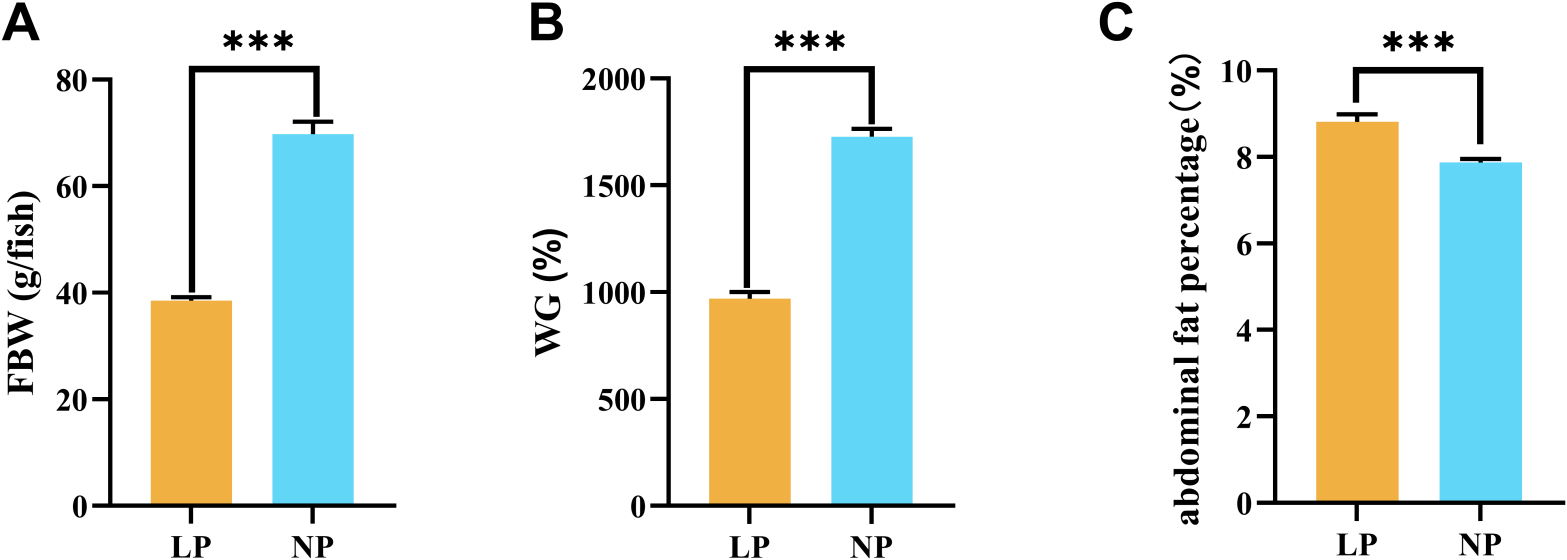
Comparative analysis of growth parameters in fish fed LP versus NP diets revealed distinct patterns in: **(A)** Final body weight; **(B)** Weight gain percentage*; **(C)** Abdominal fat percentage*. *Calculations: Weight gain (%) = [(Final - Initial body weight)/Initial body weight] × 100. Abdominal fat percentage (%) = (Abdominal fat mass/Final body weight) × 100. Data represent mean ± SEM values (n=9/group). Asterisks indicate statistically significant intergroup differences determined by two-tailed t-tests (***P < 0.001).

**Figure 4:**
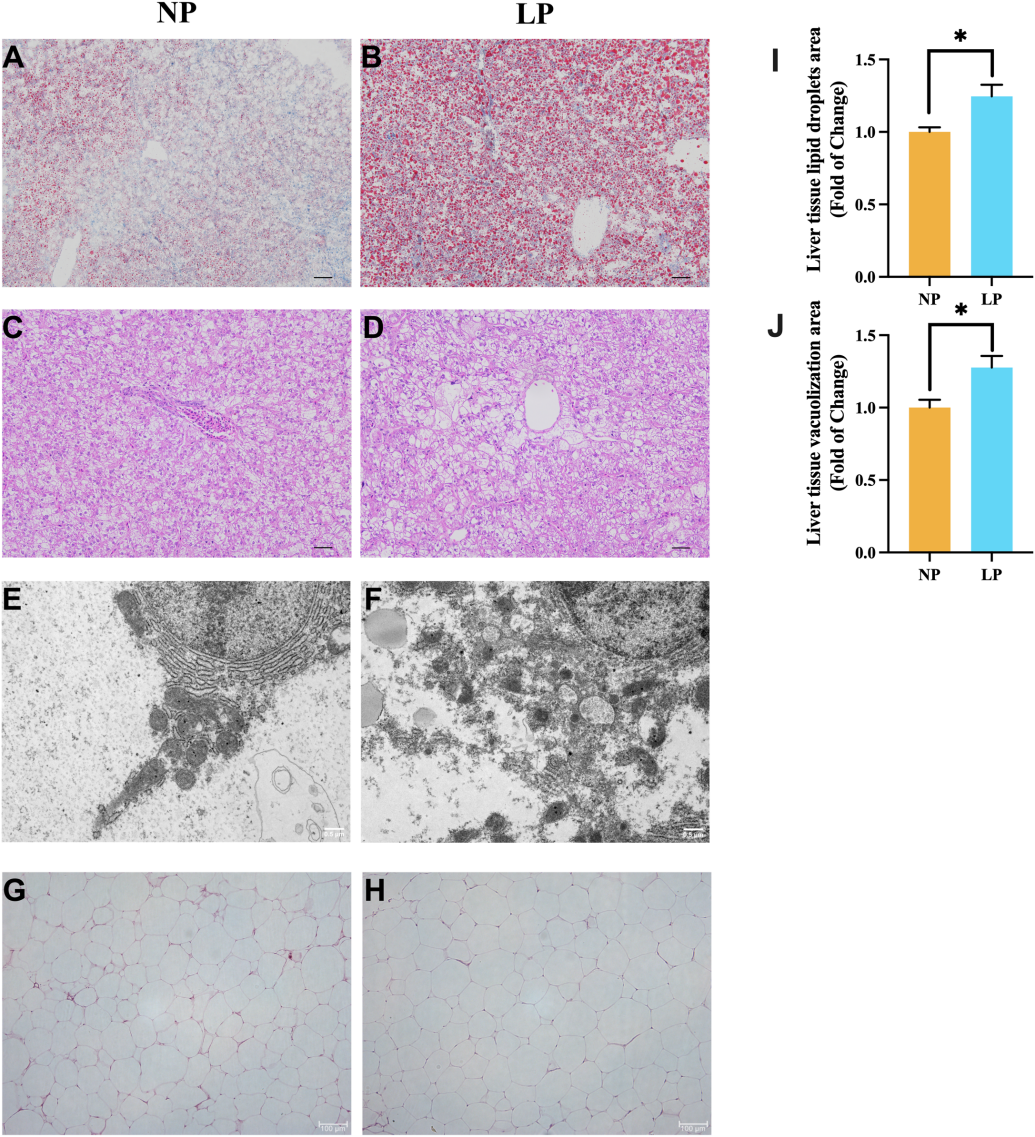
Comparative histopathological characterization of fish fed LP versus NP diet groups revealed: **(A, B)** Oil Red O-stained lipid deposition (Scale bar = 200 μm); **(C, D)** H&E-stained parenchymal architecture (Scale bar = 400 μm); **(E, F)** TEM visualization of endoplasmic reticulum (Original magnification ×7,000; Scale bar = 0.5 μm); **(G, H)** H&E-stained adipocyte morphology (Scale bar = 100 μm); **(I)** Vacuolization area of liver tissue (n = 3 fish, 3 visual fields/fish); **(J)** Lipid droplets area of liver tissue (n = 3 fish, 3 visual fields/fish). Data represent mean ± SEM (n=9/group) with asterisks indicating significance (*P < 0.05) determined by two-tailed t-tests.

Lastly, the following references were erroneously cited:

The reference for citation 15 was erroneously written as “Mazel-Sanchez B, Iwaszkiewicz J, Bonifacio JPP, Silva F, Niu C, Strohmeier S, et al. Influenza A viruses balance ER stress with host protein synthesis shutoff. Proc Natl Acad Sci U S A. (2021) 118. doi: 10.1073/pnas.2024681118”. The correct reference is “Mazel-Sanchez B, Iwaszkiewicz J, Bonifacio JPP, Silva F, Niu C, Strohmeier S, et al. Influenza A viruses balance ER stress with host protein synthesis shutoff. Proc Natl Acad Sci U S A. (2021) 118 (36): e2024681118. doi: 10.1073/pnas.2024681118”.

The reference for citation 24 was erroneously written as “Kiberstis PA. Gut microbes and obesity. Science. (2020) 368:279–80”. The correct reference is “Kiberstis PA. Gut microbes and obesity. Science. (2020) 368:279–80. doi:10.1126/science.368.6488.279-c”.

The reference for citation 89 was erroneously written as “Zhang X, Sun J, Han Z, Chen F, Lv A, Hu X, et al. Vibrio parahaemolyticus alters the community composition and function of intestinal microbiota in Pacific white shrimp, Penaeus vannamei. Aquaculture (2021) 544: 10. doi:10.1016/j.aquaculture.2021.737061. The correct reference is Zhang X, Sun J, Han Z, Chen F, Lv A, Hu X, et al. Vibrio parahaemolyticus alters the community composition and function of intestinal microbiota in Pacific white shrimp, Penaeus vannamei. Aquaculture (2021) 544: 10. doi:10.1016/j.aquaculture.2021.737061”.

There was an error in **Supplementary Table S2**, where the full names and amplification efficiencies of the primers were omitted. The corrected Supplementary Table S2 appears below.

Furthermore, many grammar and text errors are outlined below:

The 8th sentence in the **Introduction**, Paragraph 1, misstates the role of phosphorus. A correction has been made to this section. The original sentence was: “As a critical regulatory factor in lipid metabolism, phosphorus availability modulates adipose deposition patterns in fish.” The corrected sentence is: “As a critical regulatory factor in lipid metabolism, phosphorus modulates adipose deposition in fish.”

The 7th sentence in the **Introduction**, Paragraph 2, does not explicitly define the scope of “mammals.” A correction has been made to this section. The original sentence was: “Furthermore, the unfolded protein response (UPR) triggered by ER stress activates caspase-2-mediated NLRP3 inflammasome assembly (22), establishing a molecular bridge between ER stress and systemic inflammation.” The corrected sentence is: “Furthermore, in mammals, the unfolded protein response (UPR) triggered by ER stress activates caspase-2-mediated NLRP3 inflammasome assembly (22), establishing a molecular bridge between ER stress and systemic inflammation.”

The permit number was not indicated in Section 2. **Materials and methods**, Subsection *2.4 Animal Policy and Ethics*, Paragraph 1. A correction has been made to this section. The original sentence was: “The study protocol received ethical approval (2011-58) from Jimei University’s Animal Ethics Review Board, with all procedures conducted in strict accordance with established animal welfare standards.” The corrected sentence is: “The study protocol received ethical approval (Permit number: 2011-58) from Jimei University’s Animal Ethics Review Board, with all procedures conducted in strict accordance with established animal welfare standards.”

There is a lack of description of the sample collection method in Section **2. Materials and methods**, Subsection *2.4 Sample collection*, Paragraph 1. A correction has been made to this section. The original sentence was: “These samples clotted overnight at 4 °C before centrifugation (1,283 × g, 10 minutes) to isolate serum for −80 °C storage. Four jejunal samples were pooled per tank for microbiome analysis, with each specimen immediately flame-sterilized using an alcohol burner post-collection to mitigate cross-contamination risks.” The corrected sentence is: “These samples clotted overnight at 4 °C before centrifugation (1,283 × g, 10 minutes) to isolate serum for −80 °C storage. Three biological replicates were set up for each treatment in this study, and the samples from four fish were mixed together as one replicate to reduce variability between individual samples. Four jejunal samples were pooled per tank for microbiome analysis, with each specimen immediately flame-sterilized using an alcohol burner post-collection to mitigate cross-contamination risks.”

In Section 2. Materials and methods, Subsection 2.8 Statistical analysis, Paragraph 1, the “ns” in significance was not clarified, and the comparison principle of specific values was unclear. A correction has been made to this section. The original sentence was: “PICRUSt2-derived functional profiles and a-diversity indices (Shannon, Simpson) were compared between groups using Welch’s unequal variances t-test in R v4.1.2. Statistical analyses of non-microbiome data were performed using independent two-tailed t-tests in SPSS 25.0 (IBM, USA), with results presented as mean ± SEM. Significance thresholds were set at *P<0.05, **P<0.01, and ***P<0.001.” The corrected sentence is: “PICRUSt2-derived functional profiles and α-diversity indices (Shannon, Simpson) were compared between the two groups using Welch’s unequal variances t-test in R v4.1.2. Statistical analyses of non-microbiome data were performed using independent two-tailed t-tests in SPSS 25.0 (IBM, USA), with results presented as mean ± SEM. Significance thresholds were set at ns *P* ≥ 0.05, *P<0.05, **P<0.01, and ***P<0.001. In addition, the comparison of specific values in this paper follows the order of LP vs. NP, if there is no special explanation.”

The narrative of significance was not standardized in Section **3. Result**, Subsection *3.1 Growth performance and abdominal fat percentage*, Paragraph 1. A correction has been made to this section. The original sentence was: “The growth performance analysis revealed marked disparities between dietary regimens. Fish fed the LP diet attained a final body weight of 38.50 ± 0.66 g, representing a 44.8% reduction compared to fish fed the NP diet (69.78 ± 2.29 g; *P* < 0.001; **Figure 1A**).” The corrected sentence is: “Fish fed the LP diet had a final body weight of 38.50 ± 0.66 g, representing a significant reduction compared to fish fed the NP diet (69.78 ± 2.29 g) (*P* < 0.001; **Figure 1A**).”

The narrative of significance was not standardized in Section **3. Result**, Subsection *3.1 Growth performance and abdominal fat percentage*, Paragraph 1. A correction has been made to this section. The original sentence was: “This growth retardation was further corroborated by weight gain (WG) metrics, where fish fed the LP diet demonstrated 63.2% lower values relative to fish fed the NP diet (*P* < 0.001; **Figure 1B**). However, fish fed the LP diet exhibited a 2.1-fold elevation in abdominal fat percentage (3.82 ± 0.15%) versus fish fed the NP diet (1.79 ± 0.09%,*P* < 0.001; **Figure 1C**).” The corrected sentence is: “This growth retardation was further corroborated by weight gain (WG) metrics, where fish fed the LP diet (969.92 ± 17.91%) showed significantly lower values than fish fed the NP diet (1727.49 ± 21.42%) (*P* < 0.001; **Figure 1B**). However, fish fed the LP diet exhibited a significant increase in abdominal fat percentage (8.81 ± 0.10%) versus fish fed the NP diet (7.87 ± 0.05%) (*P* < 0.001; **Figure 1C**).”

The narrative of significance was not standardized in Section **3. Result**, Subsection *3.2 Phosphorus absorption and phospholipid content*, Paragraph 1. A correction has been made to this section. The original sentence was: “Serum biochemical profiling revealed distinct metabolic responses to dietary phosphorus availability. Fish fed the LP diet exhibited 58.3% higher serum phosphorus concentrations compared to fish fed the NP diet (*P* < 0.01; Figure 2A), while serum calcium levels remained stable between the two dietary groups (*P* = 0.366; Figure 2B).” The corrected sentence is: “Serum phosphorus concentrations in fish fed the LP diet (6.68 ± 0.29 mmol/L) exhibited a significant decrease compared to fish fed the NP diet (10.85 ± 0.73 mmol/L) (*P* < 0.01; Figure 2A), while serum calcium levels remained stable between the two groups (*P* = 0.366; Figure 2B).”

The significance description was not standardized, and the specific value of the measurement index was not added in Section **3. Result**, Subsection *3.2 Phosphorus absorption and phospholipid content*, Paragraph 1. A correction has been made to this section. The original sentence was: “Concurrently, alkaline phosphatase activity in fish fed the LP diet showed a 39.7% elevation relative to fish fed the NP diet (*P* < 0.05; Figure 2C).” The corrected sentence is: “Concurrently, alkaline phosphatase activity in fish fed the LP diet (17.28 ± 1.09 mmol/L) showed a significant increase compared to fish fed the NP diet (12.79 ± 0.64 mmol/L) (*P* < 0.05; Figure 2C).”

The significance description was not standardized in Section **3. Result**, Subsection *3.2 Phosphorus absorption and phospholipid content*, Paragraph 1. A correction has been made to this section. The original sentence was: “Molecular analysis of intestinal transporters demonstrated phosphorus-specific regulation, with fish fed the LP diet displaying 2.1- to 2.8-fold upregulation in *napi-iib, pit1*, and *pit2* mRNA expression compared to fish fed the NP diet (*P* < 0.05; Figure 2D). In contrast, napi-iia expression showed no dietary modulation (*P* = 0.304). Systemic phospholipid quantification identified consistent depletion patterns in fish fed the LP diet across all examined tissues.” The corrected sentence is: “Molecular analysis of intestinal transporters demonstrated phosphorus-specific regulation, with fish fed the LP diet displaying remarkable increases in *napi-iib, pit1*, and *pit2* gene expression compared to fish fed the NP diet (*P* < 0.05; Figure 2D). In contrast, *napi-iia* gene expression showed no significant difference (*P* = 0.304) between the two groups. Systemic phospholipid quantification identified consistent depletion patterns in fish fed the LP diet across all examined tissues.”

The significance description was not standardized, and the specific value of the measurement index was not added in Section **3. Result**, Subsection *3.2 Phosphorus absorption and phospholipid content*, Paragraph 1. A correction has been made to this section. The original sentence was: “Phosphoglyceride (PG) levels decreased by 36-57% and sphingomyelin (SM) by 28- 43% in serum, liver, and abdominal fat tissues compared to fish fed the NP diet, with all intergroup differences reaching statistical significance (*P* < 0.05; Figures 2E–J).” The corrected sentence is: “Phosphoglyceride (PG) and sphingomyelin (SM) contents in fish fed the LP diet were decreased in serum (PG: 71.94 ± 2.60 ng/L vs. 93.94 ± 2.60 ng/L, SM: 16.98 ± 0.78 ng/L vs. 24.00 ± 1.30 ng/L), liver (PG: 30.64 ± 0.47 ng/L vs. 37.63 ± 1.17 ng/L, SM: 41.38 ± 0.38 ng/L vs. 44.80 ± 1.14 ng/L), and abdominal fat tissues (PG: 274.12 ± 7.79 ng/L vs. 319.74 ± 12.12 ng/L, SM: 58.12 ± 2.48 ng/L vs. 72.69 ± 3.18 ng/L) compared to fish fed the NP diet, with all intergroup differences reaching statistical significance (*P* < 0.05; Figures 2E–J).”

The significance description was not standardized in Section **3. Result**, Subsection *3.3 ER stress, lipid metabolism and inflammatory response*, Paragraph 1. A correction has been made to this section. The original sentence was: “In abdominal fat tissue, fish fed the LP diet exhibited 2.3- to 3.1-fold upregulation of ER stress markers (*grp78, perk, atf6, xbp1s*) compared to fish fed the NP diet (*P* < 0.05; Figure 3A). This activation pattern extended to liver tissue, with 1.8- to 2.4-fold elevation in corresponding gene expression (*P* < 0.05; Figure 3E). Notably, ire1 mRNA expression levels remained stable in both abdominal fat tissue (*P* = 0.469) and liver (*P* = 0.774) across dietary groups.” The corrected sentence is: “In abdominal fat and liver tissues, fish fed the LP diet exhibited a remarkable upregulation of ER stress-related gene expression (*grp78, perk, atf6, xbp1s*) compared to fish fed the NP diet (*P* < 0.05; Figures 3A, E). Notably, *ire1* gene expression remained stable in both abdominal fat (*P* = 0.469) and liver (*P* = 0.774) tissues between the two groups.”

The significance description was not standardized, and the specific value of the measurement index was not added in Section **3. Result**, Subsection *3.3 ER stress, lipid metabolism and inflammatory response*, Paragraph 2. A correction has been made to this section. The original sentence was: “Specifically, triacylglycerol (TG) content in serum and liver of fish fed the LP diet showed 38-42% elevation compared to fish fed the NP diet (*P* < 0.05; Figures 3C, G). Notably, liver total cholesterol (TC) content in fish fed the LP diet reached 2.1-fold higher levels than those fed the NP diet (*P* < 0.001; Figure 3H), while serum TC concentrations remained comparable between groups (*P* = 0.970; Figure 3D).” The corrected sentence is: “Specifically, fish fed the LP diet showed a significant elevation in triacylglycerol (TG) content in serum (7.39 ± 0.20 mmol/L vs. 6.12 ± 0.08 mmol/L) and liver (0.80 ± 0.07 mmol/gpot vs. 0.55 ± 0.01 mmol/gpot) compared to fish fed the NP diet (*P* < 0.05; Figures 3C, G). Notably, liver total cholesterol (TC) content in fish fed the LP diet (0.056 ± 0.007 mmol/gpot) was significantly higher than fish fed the NP diet (0.019 ± 0.003 mmol/gpot) (*P* < 0.001; Figure 3H), while serum TC concentrations displayed no significant difference between the two groups (*P* = 0.970; Figure 3D).”

The significance description was not standardized in Section **3. Result**, Subsection *3.3 ER stress, lipid metabolism and inflammatory response*, Paragraph 2. A correction has been made to this section. The original sentence was: “Intriguingly, coordinated lipid metabolic shifts were observed across tissues. In both liver and abdominal fat tissue, fish fed the LP diet exhibited 45-62% downregulation of lipolysis genes (*pgc-1, atgl, cpt-1*), whereas lipogenesis genes (*fas, acc1, acc2*) and regulatory factors (*srebp-1, pparγ*) demonstrated 1.8- to 3.0-fold upregulation relative to fish fed the NP diet (*P* < 0.05; Figures 3B, F).” The corrected sentence is: “Intriguingly, coordinated lipid metabolic changes were observed across tissues. In both liver and abdominal fat tissue, fish fed the LP diet exhibited a remarkable downregulation of lipolysis gene expression (*pgc-1, atgl, cpt-1*), whereas the expression of lipogenesis genes (*fas, acc1, acc2*) and regulatory factors (*srebp-1, pparγ*) were remarkably upregulated compared to fish fed the NP diet (*P* < 0.05; Figures 3B, F).”

The significance description was not standardized, and the specific value of the measurement index was not added in Section **3. Result**, Subsection *3.3 ER stress, lipid metabolism and inflammatory response*, Paragraph 2. A correction has been made to this section. The original sentence was: “Nevertheless, chrebp-1 expression remained stable in both tissues (*P* = 0.598). Of particular interest, liver CHPT1 activity in the fish fed the LP diet surged to 3.2 times those fed the NP diet (*P* < 0.001; Figure 3I), contrasting sharply with unaltered EPT1 activity (*P* = 0.170; Figure 3J).” The corrected sentence is: “Nevertheless, *chrebp-1* gene expression remained stable in both tissues (*P* = 0.598). Hepatic CHPT1 activity in fish fed the LP diet (61.06 ± 1.392 ng/L) was significantly increased compared to fish fed the NP diet (52.53 ± 0.869 ng/L) (*P* < 0.001; Figure 3I), contrasting sharply with unaltered EPT1 activity (P = 0.170; Figure 3J).”

The significance description was not standardized in Section **3. Result**, Subsection *3.3 ER stress, lipid metabolism and inflammatory response*, Paragraph 3. A correction has been made to this section. The original sentence was: “Additionally, proinflammatory mediator analysis revealed tissue-specific responses. Fish fed the LP diet exhibited 2.4- to 2.8-fold upregulation of *il-1β* and *tnf-α* transcripts compared to fish fed the NP diet (*P* < 0.01). In contrast, *il-6* expression levels in abdominal fat tissue showed minimal variation between fish fed the LP diet and fish fed the NP diet (*P *= 0.433; Figure 3K).” The corrected sentence is: “Fish fed the LP diet exhibited a remarkable upregulation of *il-1β* and *tnf-α* gene expression compared to fish fed the NP diet (*P* < 0.01). In contrast, *il-6* gene expression in abdominal fat tissue showed no remarkable difference between fish fed the LP and NP diets (P = 0.433; Figure 3K).”

Inaccurate narration and wrong usage of “vs.” in Section **3. Result**, Subsection *3.4 Histology of liver and abdominal fat tissue*, Paragraph 1. A correction has been made to this section. The original sentence was: “The ORO sections revealed that the spotted seabass fed the LP diet had a greater fat accumulation in liver compared to those fed the NP diet (Figures 4A vs B, I). In H&E sections, the liver vacuolization of spotted seabass fed the LP diet was more serious than that of spotted seabass fed the NP diet (Figures 4C vs D, J).” The corrected sentence is: “The ORO sections revealed that the spotted seabass fed the LP diet had a greater fat accumulation in liver compared to fish fed the NP diet (*P* < 0.05; Figures 4A vs. B, I). In H&E sections, the vacuolization in the liver of spotted seabass fed the LP diet was more serious than that of spotted seabass fed the NP diet (*P* < 0.05; Figures 4C vs. D, J).”

Inaccurate narration and wrong usage of “vs.” in Section **3. Result**, Subsection *3.4 Histology of liver and abdominal fat tissue*, Paragraph 1. A correction has been made to this section. The original sentence was: “In addition, under the ultrastructure, it was observed that the liver endoplasmic reticulum structure of spotted seabass fed the LP diet was severely damaged, the endoplasmic reticulum was loosely stacked, and the mitochondria-associated membranes (MAMs) was disorganized (Figures 4E vs F). Meanwhile, the abdominal fat tissue of spotted seabass fed the LP diet showed adipocyte hypertrophy under H&E staining (Figures 4G vs H).” The corrected sentence is: “In addition, under the ultrastructure, the hepatocyte endoplasmic reticulum structure of spotted seabass fed the LP diet was severely damaged, the endoplasmic reticulum was loosely stacked, and the mitochondria-associated membranes (MAMs) were disorganized (Figures 4E vs. F). Meanwhile, the abdominal fat tissue of spotted seabass fed the LP diet showed adipocyte hypertrophy under H&E staining (Figures 4G vs. H).”

Inaccurate narration in Section **3. Result**, Subsection *3.5 Gut bacterial communities*, Paragraph 1. A correction has been made to this section. The original sentence was: “Microbial community analysis revealed phosphorus-dependent structural shifts. Venn diagram quantification identified 56 shared operational taxonomic units (OTUs) between groups, with fish fed the LP diet harboring 13 unique OTUs compared to 29 in fish fed the NP diet (Figure 5A).” The corrected sentence is: “Venn diagram quantification identified 56 shared operational taxonomic units (OTUs) between the two groups, with fish fed the LP diet harboring 13 unique OTUs compared to 29 in fish fed the NP diet (Figure 5A).”

The significance description was not standardized in Section **3. Result**, Subsection *3.5 Gut bacterial communities*, Paragraph 1. A correction has been made to this section. The original sentence was: “Alpha diversity metrics demonstrated significantly reduced community heterogeneity in fish fed the LP diet, exhibiting 28% lower Simpson index and 31% decreased Pielou evenness relative to fish fed the NP diet (*P* < 0.05; Figures 5B, C).” The corrected sentence is: “α-diversity metrics demonstrated significantly reduced community heterogeneity in fish fed the LP diet, exhibiting markedly lower Simpson index and markedly decreased Pielou evenness relative to fish fed the NP diet (*P* < 0.05; Figures 5B, C).”

The word “significant” was a written error in Section **3. Result**, Subsection *3.5 Gut bacterial communities*, Paragraph 1. A correction has been made to this section. The original sentence was: “Multivariate analysis confirmed distinct clustering patterns, with principal coordinates analysis (PCoA) based on Bray-Curtis distances revealing significant separation between gut microbiota profiles of fish fed the LP diet and fish fed the NP diet (*P* < 0.05; Figures 5D, E).” The corrected sentence is: “Multivariate analysis confirmed distinct clustering patterns, with principal coordinates analysis (PCoA) based on Bray-Curtis distances revealing remarkable separation between gut microbiota profiles of fish fed the LP and NP diets (*P* < 0.05; Figures 5D, E).”

The significance description was not standardized in Section **3. Result**, Subsection *3.5 Gut bacterial communities*, Paragraph 2. A correction has been made to this section. The original sentence was: “Fish fed the LP diet exhibited a 1.4-fold higher relative abundance of *Proteobacteria* (93.12% vs 66.47%) and 80.3% lower *Firmicutes* representation (6.56% vs 33.31%) compared to fish fed the NP diet (*P* < 0.05; Figures 6A, B).” The corrected sentence is: “Fish fed the LP diet exhibited markedly higher relative abundance of *Proteobacteria* (93.12% vs. 66.47%) and markedly lower *Firmicutes* representation (6.56% vs. 33.31%) compared to fish fed the NP diet (*P* < 0.05; Figures 6A, B).”

A noun plural form error occurred in Section **3. Result**, Subsection *3.5 Gut bacterial communities*, Paragraph 3. A correction has been made to this section. The original sentence was: “Potential pathogenic bacteria, such as *Plesiomonas*, were significantly more abundant (*P* < 0.05), while the abundance of potential probiotics, like *Lactococcus*, was significantly lower in fish fed the LP diet compared to those fed the NP diet (*P* < 0.05; Figure 6D).” The corrected sentence is: “Potential pathogenic bacteria, e.g., *Plesiomonas*, was significantly more abundant (*P* < 0.05), while the abundance of potential probiotics, e.g., *Lactococcus*, were significantly lower in fish fed the LP diet compared to those fed the NP diet (*P* < 0.05; Figure 6D).”

A phrase was missing after “Prevotella_7” in Section **3. Result**, Subsection *3.5 Gut bacterial communities*, Paragraph 4. A correction has been made to this section. The original sentence was: “In contrast, fish fed the NP diet had significantly enriched levels of *Lactococcus, Desulfovibrionales, Lactococcus_lactis, Bacillus, Lactobacillales, Streptococcaceae, Acidobacteriales,* and *Prevotella_7* (*P* < 0.05; LDA > 3.6; Figures 7A, B).” The corrected sentence is: “In contrast, fish fed the NP diet had significantly enriched levels of *Lactococcus*, Desulfovibrionales, *Lactococcus_lactis*, *Bacillus*, *Lactobacillales, Streptococcaceae, Acidobacteriales*, and *Prevotella_7* compared to fish fed the LP diet (*P* < 0.05; LDA > 3.6; Figures 7A, B).”

The word “normative” was a written error in Section **4. Discussion**, Paragraph 1. A correction has been made to this section. The original sentence was: “Based on our laboratory’s prior research establishing 0.72% available phosphorus (NP) as the normative dietary phosphorus level for *Lateolabrax maculatus*, whereas 0.37% available phosphorus (LP) demonstrated a significant deficiency relative to the optimal value for investigating phosphorus deprivation effects, two experimental diets were formulated accordingly (39).” The corrected sentence is: “Based on our laboratory’s prior research establishing 0.72% available phosphorus (NP) as the appropriate dietary phosphorus level for *Lateolabrax maculatus*, whereas 0.37% available phosphorus (LP) demonstrated a significant deficiency relative to the optimal value for investigating phosphorus deprivation effects, two experimental diets were formulated accordingly (39).”

The percentage before “NP” was 0.72% and should be 0.75% in Section **4. Discussion**, Paragraph 1. A correction has been made to this section. The original sentence was: “This experimental design specifically replicated these established available phosphorus concentrations (0.72% NP vs. 0.37% LP) to systematically examine phosphorus deficiency manifestations.” The corrected sentence is: “This experimental design specifically replicated these established available phosphorus concentrations (0.75% NP vs. 0.37% LP) to systematically examine phosphorus deficiency manifestations.”

A “a” was missing between “showed” and “significant” in Section **4. Discussion**, Paragraph 1. A correction has been made to this section. The original sentence was: “Serum phosphorus levels were markedly reduced in fish fed the LP diet, while alkaline phosphatase (ALP) activity showed significant elevation, a biochemical pattern aligning with observations in phosphorus-deficient teleosts (47–49).” The corrected sentence is: “Serum phosphorus levels were markedly reduced in fish fed the LP diet, while alkaline phosphatase (ALP) activity showed a significant elevation, a biochemical pattern aligning with observations in phosphorus-deficient teleosts (47–49).”

“Genes” was missing before (*napi-iib, pit1, pit2*) and “expression” was missing after (*napi-iib, pit1, pit2*) in Section **4. Discussion**, Paragraph 1. A correction has been made to this section. The original sentence was: “Molecular analysis revealed significant upregulation of intestinal sodium-phosphate cotransporters (*napi-iib, pit1, pit2*) in fish fed the LP diet compared to fish fed the NP diet, consistent with the canonical phosphorus absorption pathway mediated by Na-Pi transporters (NaPi-IIa/b/c, PIT1/2) (51).” The corrected sentence is: “Molecular analysis revealed a significant upregulation of intestinal sodium-phosphate cotransporter gene expression (*napi-iib, pit1, pit2*) in fish fed the LP diet compared to fish fed the NP diet, consistent with the canonical phosphorus absorption pathway mediated by Na-Pi transporters (NaPi-IIa/b/c, PIT1/2) (51).”

Inaccurate narration occurred in Section **4. Discussion**, Paragraph 2. A correction has been made to this section. The original sentence was: “Concurrently, ER stress markers (*grp78, perk, atf6, xbp1s*) showed significant upregulation in fish fed the LP diet versus fish fed the NP diet, suggesting phospholipid insufficiency-induced ER membrane destabilization.” The corrected sentence is: “Concurrently, ER stress-related gene expression (*grp78, perk, atf6, xbp1s*) showed a significant upregulation in fish fed the LP diet versus fish fed the NP diet, suggesting phospholipid insufficiency-induced ER membrane destabilization.”

A phrase was missing after (*pgc-1, atgl*, and *cpt-1*) in Section **4. Discussion**, Paragraph 4. A correction has been made to this section. The original sentence was: “In the current study, fish fed the LP diet exhibited higher serum TG level, increased expression of lipogenesis-related genes (*fas, acc1, acc2*), and key transcription factors of lipid metabolism (*srebp-1* and *pparγ*), along with lower expression of lipolysis-related genes (*pgc-1*, *atgl*, and *cpt-1*).” The corrected sentence is: “In the current study, fish fed the LP diet exhibited higher serum TG level, increased expression of lipogenesis-related genes (*fas, acc1, acc2*), and key transcription factors of lipid metabolism (*srebp-1* and *pparγ*), along with lower expression of lipolysis-related genes (*pgc-1, atgl*, and *cpt-1*) compared to fish fed the NP diet.”

The original expression of “between dietary groups” was not standardized in Section **4. Discussion**, Paragraph 7. A correction has been made to this section. The original sentence was: “Multivariate analysis through principal coordinates (PCoA) confirmed distinct clustering patterns between dietary groups, indicating phosphorusdependent microbiome restructuring.” The corrected sentence is: “Multivariate analysis through principal coordinates (PCoA) confirmed distinct clustering patterns between the two groups, indicating phosphorus-dependent microbiome restructuring.”

The reference numbers “90, 91” were a written error in Section **4. Discussion**, Paragraph 11. A correction has been made to this section. The original sentence was: “This microbial metabolic impairment corresponds with physiological observations, as optimal microbiota composition enhances host nutrient assimilation and metabolic efficiency (88, 89, 90, 91).” The corrected sentence is: “This microbial metabolic impairment corresponds with physiological observations, as optimal microbiota composition enhances host nutrient assimilation and metabolic efficiency (88, 89).”

The original expression of “may” was not standardized in Section **4. Discussion**, Paragraph 11. A correction has been made to this section. The original sentence was: “Notably, the reduced abundance of *Lactococcus lactis* in fish fed the LP diet versus fish fed the NP diet may compromise nutrient bioavailability, given this species’ documented capacity to upregulate intestinal growth factors and nutrient absorption mechanisms (84, 85).” The corrected sentence is: “Notably, the reduced abundance of *Lactococcus lactis* in fish fed the LP diet versus fish fed the NP diet can compromise nutrient bioavailability, given this species’ documented capacity to upregulate intestinal growth factors and nutrient absorption mechanisms (84, 85).”

There is a lack of discussion about the limitations of this study in Section **4. Discussion**, Paragraph 12. A correction has been made to this section. The original sentence was: “These collective microbial shifts likely contribute to the metabolic inefficiency and growth retardation observed under phosphorus restriction.” The corrected sentence is: “These collective microbial shifts likely contribute to the metabolic inefficiency and growth retardation observed under phosphorus restriction. Although this study identified phospholipid synthesis limitations caused by low phosphorus levels, it still lacks in-depth exploration of specific aspects such as the exact affected types of phospholipids and the precise mechanisms triggering endoplasmic reticulum (ER) stress. Future experiments will further investigate the specific phospholipid changes induced by low phosphorus and the regulated mechanisms in ER.”

An extra “and” was mistakenly written between “phospholipid” and “in” in Section **5. Conclusion**, Paragraph 1. A correction has been made to this section. The original sentence was: “In this study (Figure 8), LP led to the decreased content of phospholipid and in spotted seabass, which in turn induced ER stress, disturbed lipid metabolism and inflammatory response.” The corrected sentence is: “In this study (Figure 8), LP led to the decreased content of phospholipid in spotted seabass, which in turn induced ER stress, disturbed lipid metabolism and inflammatory response.”

The original version of this article has been updated.

