# Supplementary material for: Dietary phosphorus restriction induced phospholipid deficiency, endoplasmic reticulum stress, inflammatory response and gut microbiota disorders in Lateolabrax maculatus
Source: Front Immunol. 2025 May 15;16:1592806. doi: 10.3389/fimmu.2025.1592806 (PMC12119276; doi:10.3389/fimmu.2025.1592806)
Supplement: Supplementary file 1 [file Table1.docx]

**Table S1**

Formulation and proximate composition of the experimental diets (dry weight, %).

| **Ingredients (%)** |  | **Treatment Groups** | | |  | |
| --- | --- | --- | --- | --- | --- | --- |
|  |  | **LP** | | **NP** | | |
| Deboned fishmeal |  | 15.00 | | 15.00 | | |
| Squid paste |  | 3.00 | | 3.00 | | |
| Casein |  | 22.00 | | 22.00 | | |
| Wheat gluten |  | 8.00 | | 8.00 | | |
| Dextrin |  | 35.00 | | 35.00 | | |
| Microcrystalline cellulose |  | 3.52 | | 2.91 | | |
| Fish oil |  | 7.00 | | 7.00 | | |
| Soybean oil |  | 3.00 | | 3.00 | | |
| Vitamin premix^a^ |  | 0.40 | | 0.40 | | |
| Mineral premix^b^ |  | 0.50 | | 0.50 | | |
| Vitamin C |  | 0.10 | | 0.10 | | |
| Choline chloride |  | 0.50 | | 0.50 | | |
| NaH_2_PO_4_ |  | 0.00 | | 0.98 | | |
| K_2_HPO_4_ |  | 0.00 | | 0.69 | | |
| KCl |  | 1.00 | | 0.41 | | |
| NaCl |  | 0.78 | | 0.30 | | |
| Taurine |  | 0.10 | | 0.10 | | |
| Y_2_O_3_ |  | 0.10 | | 0.10 | | |
| **Proximate compositions (%)** |  |  |  | | |  |
| Crude protein |  | 45.76 | | 45.76 | | |
| Crude lipid |  | 12.43 | | 12.43 | | |
| Available phosphorus |  | 0.37 | | 0.75 | | |
| Total calcium |  | 0.11 | | 0.11 | | |
| Total chlorine |  | 0.95 | | 0.38 | | |

NaH_2_PO_4_, sodium dihydrogen phosphate; K_2_HPO_4_, dipotassium hydrogen phosphate; KCl, potassium chloride; NaCl, sodium chloride; Y_2_O_3_, yttrium (III) oxide.

^a,b^ The mineral and vitamin premixes were prepared following the method described in our previous work. (Zhang et al., 2015).
