# Supplementary material for: Dietary phosphorus restriction induced phospholipid deficiency, endoplasmic reticulum stress, inflammatory response and gut microbiota disorders in Lateolabrax maculatus
Source: Front Immunol. 2025 May 15;16:1592806. doi: 10.3389/fimmu.2025.1592806 (PMC12119276; doi:10.3389/fimmu.2025.1592806)
Supplement: Supplementary file 2 [file Table2.docx]

Table S2

Sequences of primer used in real-time PCR.

| Gene | Forward (5′ → 3′) | Reverse (5′ → 3′) |
| --- | --- | --- |
| *β-actin* | CAACTGGGATGACATGGAGAAG | TTGGCTTTGGGGTTCAGG |
| *napi-iia* | GAAGAGGATAAGTGGGGAGACG | AAATGGAGGCTGAAGCGAAG |
| *napi-iib* | GGTGTTAGTCACAGTGGGGG | GGTGACAGACGTTCCGATGT |
| *pit1* | CTGCTTCCTCCCCATCAACA | GGATAACCCACACACCAAACCA |
| *pit2* | ATCACGAGGAGAAGGATAAGCC | CATTACACCGCCCTGGTCATA |
| *pgc-1* | GTTCCTCCGAACTCCCAGTG | GCAACACCCCTCCAACTACA |
| *atgl* | CTTCCTCTCCGCAACAAGTC | TGGTGCTGTCTGGAGTGTTC |
| *cpt-1* | CCTCAATGATACATCGGAACCC | CTGCGGCTCATCATCTAACG |
| *fas* | AAACTGAAGCCCTGTGTGCC | CACCCTGCCTATTACATTGCTC |
| *acc1* | AAGGCGGTGGTGATGGATTT | GGCCATGTCGCCTTTGTTTT |
| *acc2* | CTGTCCGCCTGTTTCTCACT | TGCAGCAGACCCTGCTTATC |
| *chrebp* | GTGACAACGCTCAGCTCTCA | TGATGGCAGAGTTCAGGAGC |
| *srebp-1c* | CCTCACTCTGCAGCCAATCA | CGTAGTCCCACCCTCAAACC |
| *pparγ* | CAGTGGCAGCGTTAAACATCG | GAAGAAACCCTTACAGCCCTCA |
| *grp78* | GGGAGAAGAGGAGAAAGGTCT | GCTTATCACCGCTCCGCTT |
| *ire1* | AAAGGTTGTTCAGGGTGGCAT | GCAGCAATCAATCAACAAGCAAA |
| *atf6* | AACGAGCACTTGAGGAGAGC | CAGACGCTCGCCCCTGTTA |
| *perk* | GTTTTCACCCCAGCAAGCAG | AACCTTAGTGTCGGCCTTGG |
| *xbp1s* | CTACTGACAGAAAATGAGGAAC | GCAAATCAGACTCACTGTCTG |
| *il-6* | TACAATGTCCTCCTCAAGCACG | GCCTTTGACCTCCTCCATCAG |
| *tnf-α* | GACTCCATAGGCAGCAAAGC | AGAAAGTCTTGCCCTCGTCA |
| *il-1β* | CATAGGGATGGGGACAACGA | AGGGGACGGACACAAGGGTA |

*napiiia,* sodium-phosphate cotransporter iia; *napiiib*, sodium-phosphate cotransporter iib; *pit1*, sodium-phosphate cotransporter iic 1; *pit2*, sodium-phosphate cotransporter iic 2; *pgc-1*, peroxisome proliferator-activated receptor-gamma coactivator 1alpha; *atgl*, adipose triglyceride lipase; *cpt-1*, carnitine palmitoyltransferase 1; *fas*, fatty acid synthetase; *acc1*, acetyl-coa carboxylase 1; *acc2*, acetyl-coa carboxylase 2; *chrebp*, carbohydrate response element binding protein; *srebp-1c*, sterol-regulatory element binding proteins 1c; *pparγ*, peroxisome proliferator-activated receptor γ; *grp78*, glucose-regulated protein 78; *ire1*, inositol requiring enzyme 1; *atf6*, activating transcription factor 6; *perk*, dsRNA-activated protein kinase-like ER kinase; *xbp1s*, X-box binding protein 1; *il-6*, interleukin-6; *tnf-α*, tumor necrosis factor α; *il-1β*, Interleukin-1β.
